# VIM-encoding Inc_pSTY_ plasmids and chromosome-borne integrative and mobilizable elements (IMEs) and integrative and conjugative elements (ICEs) in *Pseudomonas*

**DOI:** 10.1186/s12941-022-00502-w

**Published:** 2022-03-09

**Authors:** Fangzhou Chen, Peng Wang, Zhe Yin, Huiying Yang, Lingfei Hu, Ting Yu, Ying Jing, Jiayao Guan, Jiahong Wu, Dongsheng Zhou

**Affiliations:** 1 State Key Laboratory of Pathogen and Biosecurity, Beijing Institute of Microbiology and Epidemiology, Beijing, 100071 China; 2 Basic Medical College, Guizhou Medical University, Guiyang, 550025 China; 3 Guangzhou Medical University, Guangzhou, 511436 China

**Keywords:** *Pseudomonas*, *Bla*_VIM_, Mobile genetic elements, Integrons, Antibiotic resistance, ST129

## Abstract

**Background:**

The carbapenem-resistance genes *bla*_VIM_ are widely disseminated in *Pseudomonas*, and frequently harbored within class 1 integrons that reside within various mobile genetic elements (MGEs). However, there are few reports on detailed genetic dissection of *bla*_VIM_-carrying MGEs in *Pseudomonas*.

**Methods:**

This study presented the complete sequences of five *bla*_VIM-2/-4_-carrying MGEs, including two plasmids, two chromosomal integrative and mobilizable elements (IMEs), and one chromosomal integrative and conjugative element (ICE) from five different *Pseudomonas* isolates.

**Results:**

The two plasmids were assigned to a novel incompatibility (Inc) group Inc_pSTY_, which included only seven available plasmids with determined complete sequences and could be further divided into three subgroups Inc_pSTY_-1/2/3. A detailed sequence comparison was then applied to a collection of 15 MGEs belonging to four different groups: three representative Inc_pSTY_ plasmids, two Tn*6916*-related IMEs, two Tn*6918*-related IMEs, and eight Tn*6417*-related ICEs and ten of these 15 MGEs were first time identified. At least 22 genes involving resistance to seven different categories of antibiotics and heavy metals were identified within these 15 MGEs, and most of these resistance genes were located within the accessory modules integrated as exogenous DNA regions into these MGEs. Especially, eleven of these 15 MGEs carried the *bla*_VIM_ genes, which were located within 11 different concise class 1 integrons.

**Conclusion:**

These *bla*_VIM_-carrying integrons were further integrated into the above plasmids, IMEs/ICEs with intercellular mobility. These MGEs could transfer between *Pseudomonas* isolates, which resulted in the accumulation and spread of *bla*_VIM_ among *Pseudomonas* and thus was helpful for the bacteria to survival from the stress of antibiotics. Data presented here provided a deeper insight into the genetic diversification and evolution of VIM-encoding MGEs in *Pseudomonas*.

**Supplementary Information:**

The online version contains supplementary material available at 10.1186/s12941-022-00502-w.

## Introduction

VIM hydrolyzes nearly all β-lactam except for aztreonam and currently consists of 76 variants (https://www.ncbi.nlm.nih.gov/pathogens/refgene) [1]. The *bla*_VIM_ genes are mostly found in *Pseudomonas* and Enterobacteriaceae and occur frequently within class 1 integrons with distinct gene cassette arrays (GCAs). The *bla*_VIM_-carrying integrons are always presented in various mobile genetic elements (MGEs) such as plasmids, integrative and conjugative elements (ICEs), and integrative and mobilizable elements (IMEs), enhancing the mobility and dissemination of *bla*_VIM_ genes [2].There are a wealth of sequenced *bla*_VIM_-carrying plasmids, which can be assigned to various incompatibility (Inc) groups such as IncA, IncC, IncN, IncP and IncL/M [3–7]. IMEs/ICEs are frequently located in bacterial chromosomes: ICEs encode self-conjugation modules and thus are able to transfer between cells [8], whereas IMEs needs a helper conjugative element to achieve intercellular mobility [9].

Our previous studies showed the detailed genetic characteristics of two novel carbapenemase-encoding MGEs: SIM-encoding plasmid pHN39-SIM [10] and *bla*_VIM-4_-containing ICE Tn*6413* [7]. This follow-up study presented the complete sequences of five novel *bla*_VIM-2/-4_-carrying MGEs, including two plasmids pJ20133-VIM and p716811-VIM, and two chromosomal IMEs Tn*6917* and Tn*6918*, and one chromosomal ICE Tn*6953* from *Pseudomonas* isolates. These two plasmids were assigned to a novel Inc group Inc_pSTY_. Comprehensive sequence comparisons were then applied to three representative Inc_pSTY_ plasmids and 12 chromosome-borne IMEs and ICEs (including the above five ones), providing a deeper understanding of the genetic diversification and evolution of VIM-encoding MGEs in *Pseudomonas*.

## Materials and methods

### Bacterial strains and identification

Five clinical isolates *P. aeruginosa* SE5443, *P. putida* 716811 and 159349, and *P. monteilii* J20133 and 918607 were collected from four different Chinese public hospitals (Additional file 1: Table S1). The 16S rRNA genes and the *bla*_VIM_ genes were detected as described previously [7].

### Genomic DNA extraction, sequencing, and sequence assembly

Bacterial genomic DNA was isolated using the UltraClean Microbial Kit (Qiagen, NW, Germany), and sequenced from a sheared DNA library with average size of 15 kb (ranged from 10 to 20 kb) on a PacBio RSII sequencer (Pacific Biosciences, CA, USA), as well as a paired-end library with an average insert size of 350 bp (ranged from 150 to 600 kb) on a HiSeq sequencer (Illumina, CA, USA) [11]. The paired-end short Illumina reads were used to correct the long PacBio reads utilizing proovread [12], and then the corrected PacBio reads were assembled de novo utilizing SMARdenovo (https://github.com/ruanjue/smartdenovo).

### Sequence annotation and comparison

Genome sequences were annotated by the Rapid Annotation using Subsystem Technology (RAST) [13] combined with BLASTP/BLASTX/BLASTN [14], Domain (https://www.ncbi.nlm.nih.gov/Structure/cdd/wrpsb.cgi), and RefSeq database [15]. Annotation of resistance genes, MGEs, and other features were carried out using the online databases including CARD [16], ResFinder [17], ISfinder [18], INTEGRALL [19], and Tn Number Registry [20]. Multiple and pairwise sequence comparisons were performed using BLASTN. Gene organization diagrams were drawn using Inkscape 1.0 (https://inkscape.org/en/).

### Phylogenetic analysis

Indicated nucleotide sequences were aligned using Clustal Omega 1.2.2 [21], and then maximum-likelihood phylogenetic trees were constructed from aligned sequences using MEGA X 10.1.8 [22] with a bootstrap iteration of 1000.

### Conjugal transfer

Each indicated *bla*_VIM_-carrying MGE was transformed from its wild-type isolate into rifampin-resistant *P. aeruginosa* ATCC 27853 or PAO1, through conjugal transfer or electroporation experiments. Three milliliters of overnight cultures of each donor and recipient bacteria were mixed together, harvested, and resuspended in 80 mL of Brain Heart Infusion (BHI) broth (BD Biosciences). The mixture was spotted on a 1 cm^2^ hydrophilic nylon membrane filter with a 0.45 µm pore size (Millipore) that was placed on BHI agar (BD Biosciences) plate and then incubated for mating at 26 °C or 37 °C for 12 h to 18 h. Bacteria were washed from filter membrane and spotted on BHI plates, for selecting a *bla*_VIM_-carrying transconjugant. 1500 mg/mL rifampin (for ATCC 27853 or PAO1), together with 4 mg/mL meropenem (for *bla*_VIM_) was used as transconjugant selection.

### Phenotypic assays and multi-locus sequence typing (MLST)

Bacterial antimicrobial susceptibility was tested by BioMérieux VITEK 2, and interpreted as per the 2020 Clinical and Laboratory Standards Institute (CLSI) guidelines [23]. Activity of Amber class A/B/D carbapenemases was determined with a modified CarbaNP test [24]. The sequence types (STs) of *Pseudomonas* isolates were identified according to the online *Pseudomonas* MLST schemes (https://pubmlst.org/organisms).

## Results

### Identification of three STs for *Pseudomonas* isolates

Three different STs, namely ST129, ST17, and ST639, were identified from the five *Pseudomonas* isolates (Additional file 1: Table S1), whose complete genome sequences were determined in this work. *P. putida* 716811 and 159349 belonged to ST129 and ST17, respectively. *P. aeruginosa* SE5443 belonged to ST639. ST129 was a novel ST of *P. putida*. The two *P. monteilii* isolates J20133 and 918607 could not be assigned with any ST types due to lack of *P. monteilii* MLST scheme.

### Proposal of a novel group of Inc_pSTY_ plasmids

**Fig. 1:**
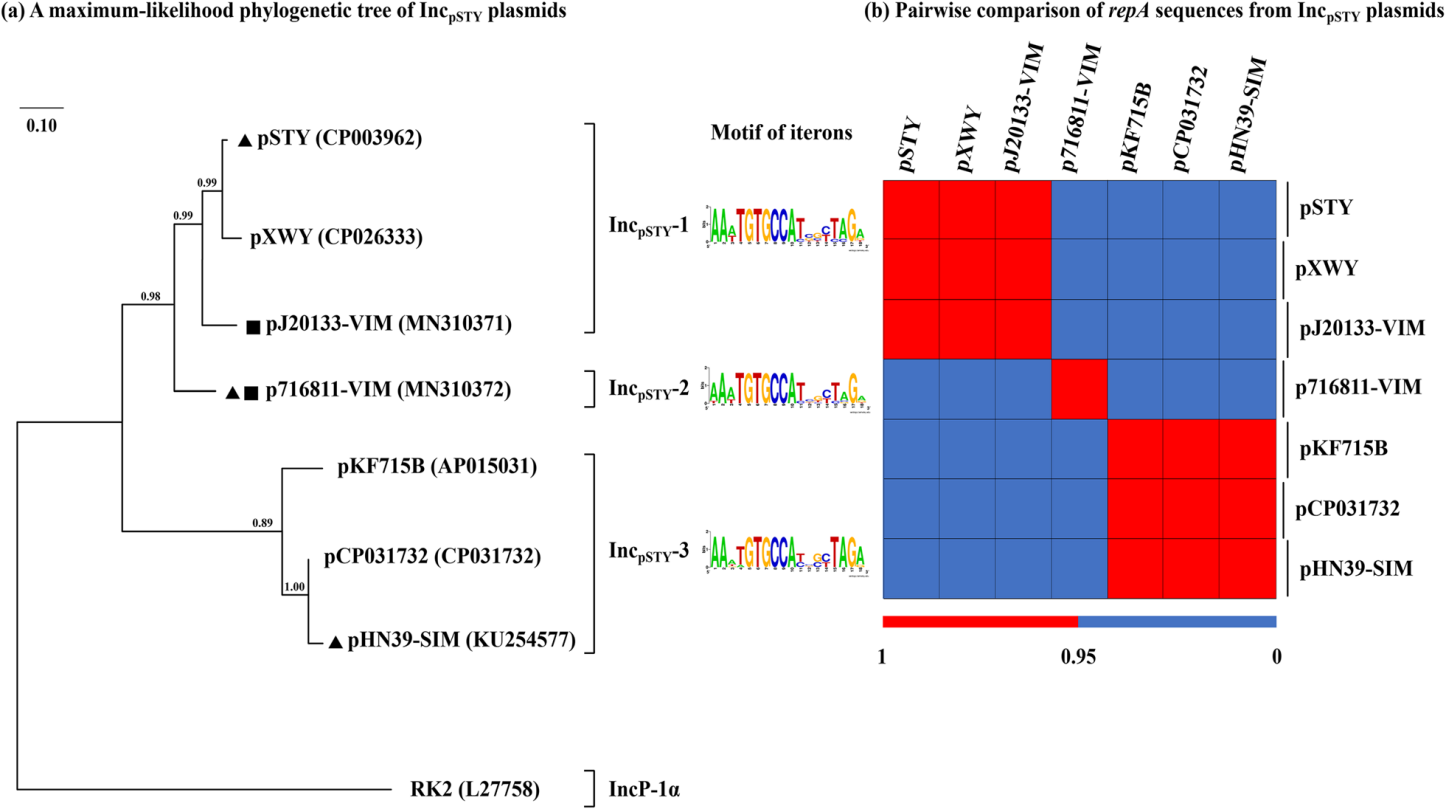
Evolutionary relationships of the seven Inc_pSTY_ plasmids. **a** A maximum likelihood phylogenetic tree is constructed from aligned *repA* sequences. IncP-1α plasmid RK2 [60] is used as the outgroup. Degree of support (percentage) for each cluster of associated taxa, as determined by bootstrap analysis, is shown next to each branch. Bar corresponds to scale of sequence divergence. Triangle indicates the reference plasmid of each Inc_pSTY_ subgroup, whilst squares denote the plasmids fully sequenced in this study. **b** A heatmap of pairwise comparison of *repA* sequences. See Table S3 for original BLAST coverage and nucleotide identity values

**Fig. 2:**
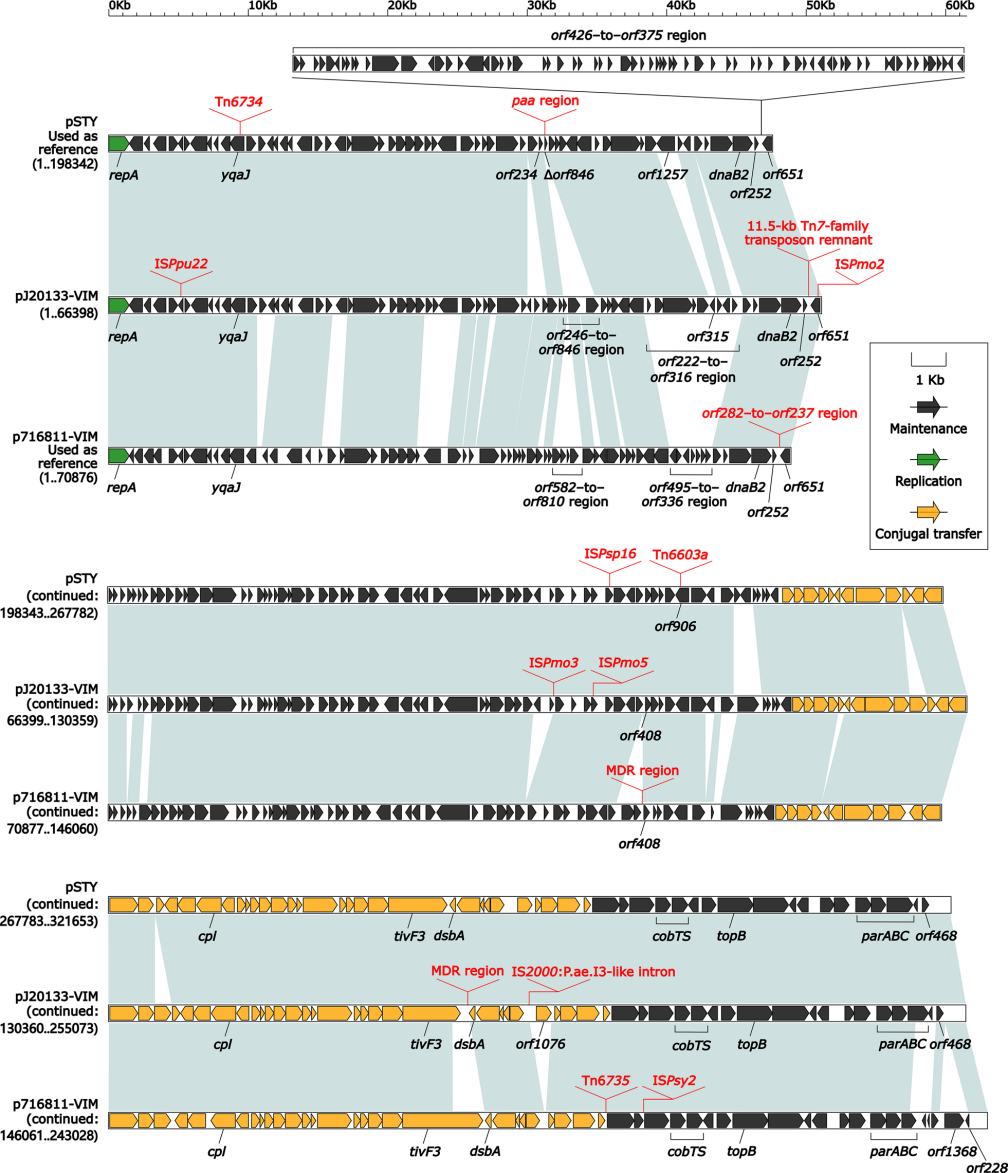
Linear comparison of the three Inc_pSTY_ plasmids pSTY, pJ20133-VIM, and p716811-VIM. Genes are denoted by arrows. Genes, mobile elements and other features are colored based on function classification. Light blue shading regions denote nucleotide identity > 80%. Red characters represent accessory modules. Numbers in brackets indicate nucleotide positions within corresponding plasmids

Two *bla*_VIM_-carrying plasmids pJ20133-VIM and p716811-VIM were identified from the complete genome sequences of the two *Pseudomonas* isolates J20133 and 716811 (Additional file 1: Table S1). A novel Inc_pSTY_ group was proposed from a total of seven available fully sequenced single-replicon plasmids (Additional file 1: Table S2; last accessed August 10th 2020) that included the above two plasmids together with five additional ones from GenBank, because these seven plasmids harbored not only homologous *repA* (replication initiation protein) genes together with its iterons (Fig. 1) but also similar backbone gene organizations (Fig. 2). Since the Inc group of plasmids could be divided according to the homology of *repA* genes [25–27], a phylogenetic tree (Fig. 1) was constructed based on the *repA* sequences of these seven plasmids, showing that these seven plasmids could be divided into three separately clustering subgroups Inc_pSTY_-1/2/3. As shown by pairwise comparison of *repA* sequences, plasmids within each subgroup showed ≥ 95% identity, while those from different subgroups displayed ≤ 95% but ≥ 82% identity (Additional file 1: Table S3). pSTY [28], p716811-VIM, and pHN39-SIM [10] were the first sequenced plasmids of Inc_pSTY_-1, Inc_pSTY_-2, and Inc_pSTY_-3, respectively, and thus were identified as the references of the corresponding Inc_pSTY_ subgroups.

### Comparison of three Inc_pSTY_ plasmids pSTY, pJ20133-VIM and p716811-VIM

A detailed comparative genomics analysis was applied to three representative Inc_pSTY_ plasmids including the Inc_pSTY_-1 plasmids pSTY and pJ20133-VIM and the Inc_pSTY_-2 plasmid p716811-VIM. The Inc_pSTY_-3 plasmid pHN39-SIM was not included herein because it had been detailed characterized in our previous study [10]. These three characterized plasmids varied in size from about 243 to 321 kb with predicted ORFs from 255 to 282 (Table 1). The modular structure of each plasmid was divided into the backbone, and the accessory modules that were acquired DNA regions and inserted at different sites of the backbone (Fig. 2). pSTY, pJ20133-VIM, and p716811-VIM had > 80% nucleotide identity across > 66% of their backbone sequences, while pSTY and pJ20133-VIM shared > 74% of their backbones with > 95% nucleotide identity (Additional file 1: Table S4). These three plasmids shared the key Inc_pSTY_ backbone markers: *repA* together with its iterons, *parABC* for partition, and multiple conjugal transfer genes (Fig. 2). A 47.9-kb backbone region *orf426*–to–*orf375* was presented in pSTY, but it was deleted from pJ20133-VIM and p716811-VIM due to insertion of an 11.5-kb Tn*7*-family transposon remnant (see below) and a 21.6-kb *orf282*–to–*orf237* region, respectively, at the same backbone site. The *orf282* − to − *orf237* region carried IS*1491*, *mvaT* (DNA-binding domain of bacterial xenogeneic silencer MvaT), five unnamed genes (encoding nucleoid-associated protein, predicted NTPase, AAA family ATPase, endonuclease, and putative transposase, respectively), and eight genes encoding hypothetical proteins.

**Table 1:** Major features of the three Inc_pSTY_ plasmids analyzed

Plasmid	Accession number	Total length (bp)	Total number of ORFs	Mean G + C content (%)	Length of backbone (bp)	Accessory modules		*bla*_VIM_	Host bacterium
						Resistance	Non-resistance		
pSTY	CP003962	321,653	355	56.8	210,612	*paa* region	IS*Psp16*, Tn*6734*, and Tn*6603a*	None	*Pseudomonas taiwanensis* VLB120
pJ20133-VIM	MN310371	255,073	298	56.5	168,325	MDR region	IS*Ppu22*, IS*Pmo2*, IS*Pmo3*, IS*Pmo5*, IS*2000*:P.ae.I3-like intron, and 11.5-kb Tn*7*-family transposon remnant	*bla*_VIM-2_	*P. monteilii* J20133
p716811-VIM	MN310372	243,028	262	55.4	163,184	MDR region	IS*Psy2*, *orf282* − to − *orf237* region, and Tn*6735*	*bla*_VIM-2_	*P. putida* 716,811

The Inc_pSTY_-1 reference plasmid pSTY [28] from GenBank, and the Inc_pSTY_-1 plasmid pJ20133-VIM plus the Inc_pSTY_-2 reference plasmid p716811-VIM sequenced in this study were included in a detailed sequence comparison

pSTY, pJ20133-VIM, and p716811-VIM had totally different profiles of accessory modules (Table 1 and Fig. 2): (i) a 59.7-kb *paa* (phenylacetic acid degradation) region carrying *paa* locus and *mer* (mercury resistance) locus, Tn*6734*, Tn*6603a*, and IS*Psp16* in pSTY; (ii) a 67.2-kb MDR (multi-drug resistance) region, the 11.5-kb Tn*7*-family transposon remnant, IS*2000*:P.ae.I3-like intron, IS*Pmo2*, IS*Ppu22*, IS*Pmo3*, and IS*Pmo5* in pJ20133-VIM; and (iii) a 29.6-kb MDR region, Tn*6735*, *orf282*–to–*orf237* region, IS*1491*, and IS*Psy2* in p716811-VIM.

The integration of the 59.7-kb *paa* region into pSTY led to truncation of *orf246*–to–*orf846* region (as originally observed in pJ20133-VIM) into a 138-bp *orf846* remnant (Fig. 2). A presumed prototype *paa* region was terminally bracketed by two copies of Tn*5563* [29]; the right copy was intact while the left copy was truncated due to insertion of Tn*6481* in the 59.7-kb *paa* region from pSTY (Additional file 1: Fig. S1). This 59.7-kb *paa* region still contained a complete set of phenylacetic acid degradation genes (Additional file 1: Fig. S1). Tn*6734* was an IME containing a phenol degradation gene locus (Additional file 1: Fig. S2a). Tn*6603a* was a Tn*3*-family unit transposon containing an oxidative stress defense gene *osmC* (Additional file 1: Fig. S2b). The 67.2-kb MDR region from pJ20133-VIM contained In58, Tn*5046* and ΔTn*512*, a 3.6-kb Tn*4662a* remnant, and several IS elements (Additional file 1: Fig. S3).

**Fig. 3:**
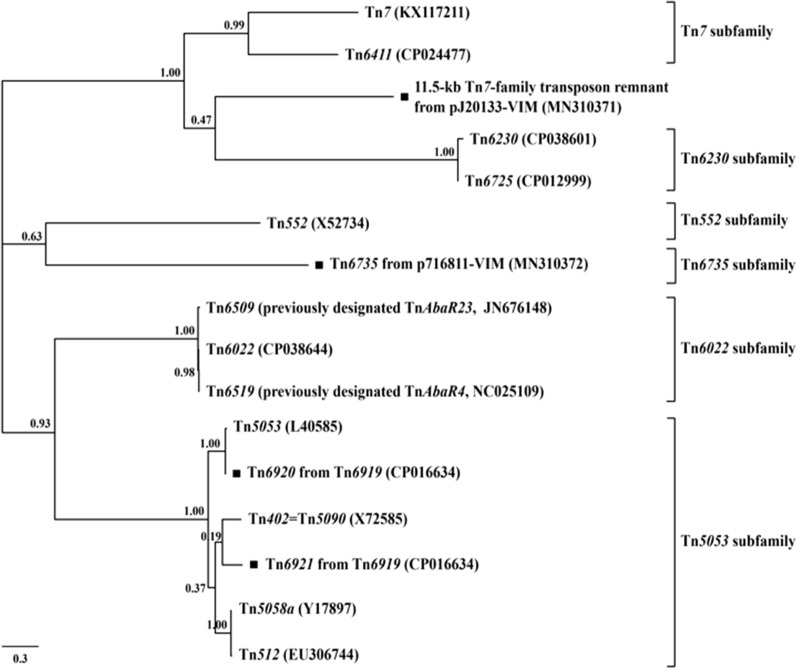
Evolutionary relationships of the Tn*7*-family transposons. A maximum likelihood phylogenetic tree was constructed from aligned *tnsA* sequences. Degree of support (percentage) for each cluster of associated taxa, as determined by bootstrap analysis, is shown next to each branch. Bar corresponds to scale of sequence divergence. Squares denote Tn*7*-family transposons or transposon remnants sequenced in this study

According to the previous grouping scheme of Tn*7*-family transposons [30], the phylogenetic tree was constructed based on the nucleotide sequences of *tnsA*, which encoded the endonuclease responsible for excision of Tn*7*-family transposons. A phylogenetic analysis of Tn*6735*, the 11.5-kb Tn*7*-family transposon remnant, Tn*6922*, and Tn*6921*, together with additional 12 representative sequenced Tn*7*-family transposons from GenBank based on the *tnsA* genes, indicated that the first two belonged to a novel Tn*6735* subfamily and an unnamable subfamily (it had no intact sequenced transposon), respectively (Fig. 3). Similar to Tn*7* [31], both Tn*6735* (carrying pyrimidine biosynthesis genes) and the 11.5-kb Tn*7*-family transposon remnant encoded the core transposition determinants (endonuclease TnsA, transposase TnsB, regulator TnsC, target-site selection protein TnsD, and TnsB-binding sites), and the later one still encoded an additional target-site selection protein TnsE (Additional file 1: Fig. S4).

The 29.6-kb MDR region from p716811-VIM has a primary structure of a P.p.I3-like intron with interruption by Tn*6740* that carried *bla*_VIM-2_, *aacA4*, *qnrVC1*, and *mer* locus (Additional file 1: Fig. S5). As a derivant of the IS*66*-family transposon Tn*5042* [32], Tn*6740* retained the whole Tn*5042* sequence with integration of a 21.2-kb region that comprised *mer*-containing Tn*3*-family unit transposon Tn*5563* [29], *qnrVC1*, and *bla*_VIM-2_-carrying In1722 (see below) (Additional file 1: Fig. S5).

### Collection of 12 chromosome-borne IMEs/ICEs for sequence comparison

Three *bla*_VIM_-carrying chromosomal MGEs Tn*6917*, Tn*6918* and Tn*6953* were identified from the complete genome sequences of the three *Pseudomonas* isolates 918607, 159349, and SE5443 (Additional file 1: Table S1). A detailed sequence comparison was applied to a total of 12 chromosomal MGEs belonging to three groups: two related IMEs Tn*6916* and Tn*6917*; two related IMEs Tn*6918* and Tn*6919*; and eight related ICEs Tn*6417*, Tn*6413*, Tn*6953*, Tn*6454*, Tn*6455*, Tn*6456*, Tn*6457* and Tn*6458* (Table 2; last accessed August 10th 2020). Tn*6917* was the only *bla*_VIM_-carrying one belonging to Tn*6916*-related IMEs in GenBank. Tn*6919* was the only one (in addition to Tn*6918*) belonging to Tn*6918*-related IMEs in GenBank, and it did not carry *bla*_VIM_. The seven Tn*6417*-related ICEs were selected from GenBank because Tn*6417* was used as reference and the rest six ones Tn*6413*, Tn*6954*, Tn*6955*, Tn*6956*, Tn*6957* and Tn*6958* carried *bla*_VIM_ genes. Similar to the above Inc_pSTY_ plasmids, the modular structure of each IME/ICE was divided into the backbone and the accessory modules.

**Table 2:** Major features of the 12 chromosome-borne IMEs and ICEs analyzed

Category	MGE	Accession number	Host bacterium	Country	*bla*_VIM_	Nucleotide position in the chromosome	Refs.
Tn*6916*-related IMEs	Tn*6916*	CP014062	*P. monteilii* FDAARGOS_171	USA	None	Not applicable	[33]
	Tn*6917*	CP043395	*P. monteilii* 918,607	China	*bla*_VIM-2_	2624899..2625699	This study
Tn*6918*-related IMEs	Tn*6918*	CP045553	*P. putida* 159,349	China	*bla*_VIM-2_	813783..814583	This study
	Tn*6919*	CP016634	*P. putida* IEC33019	Brazil	None	Not applicable	—
Tn*6417*-related ICEs	Tn*6417*	CP013993	*P. aeruginosa* DHS01	France	None	Not applicable	[7]
	Tn*6413*	CP030075	*P. aeruginosa* 6762	China	*bla*_VIM-4_	409196..409996	[7]
	Tn*6953*	CP046405	*P. aeruginosa* SE5443	China	*bla*_VIM-2_	4906548..4907348	This study
	Tn*6954*	KY623658	*Alcaligenes faecalis* GZAF3	Palestine	*bla*_VIM-2_	63643..64443	[36]
	Tn*6955*	MF168944	*P. aeruginosa* HSV3483	Portugal	*bla*_VIM-2_	62615..63415	[3]
	Tn*6956*	MF168946	*P. aeruginosa* FFUP_PS_CB5	Portugal	*bla*_VIM-2_	93931..94731	[3]
	Tn*6957*	CP043328	*P. aeruginosa* CCUG 51971	Sweden	*bla*_VIM-4_	5251865..5252665	–
	Tn*6958*	CP048039	*A. faecalis* MUB14	Poland	*bla*_VIM-4_	659107..659907	[37]

A collection of 12 fully sequenced chromosome-borne MGEs, which included all the four Tn*6916*- or Tn*6918*-related IMEs and the eight Tn*6417*-related ICEs composed of Tn*6417* itself plus all the six *bla*_VIM_-carrying ones, were included in a detailed sequence comparison

### Comparison of two related IMEs Tn*6916* and Tn*6917*

**Fig. 4:**
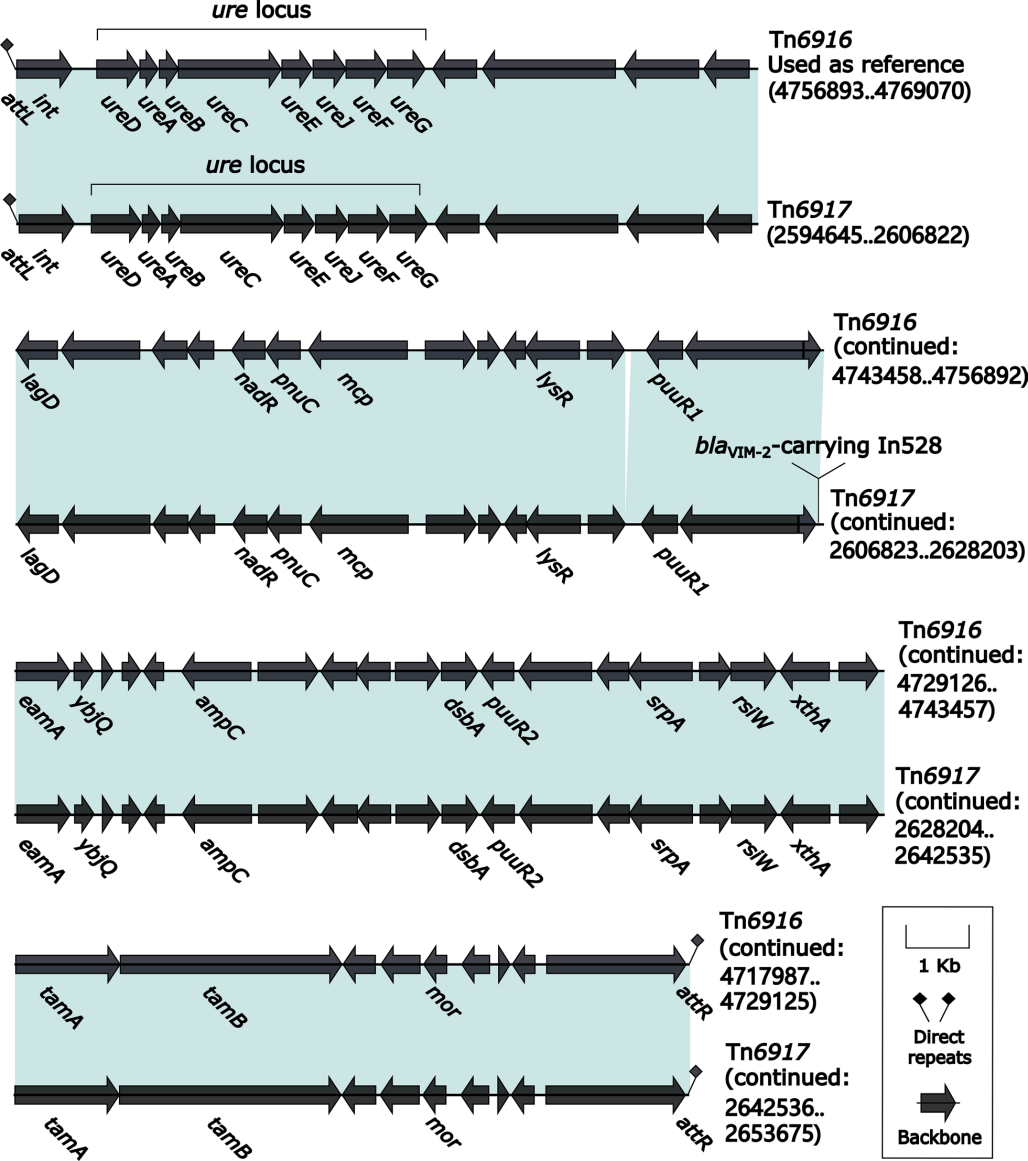
Comparison of the two-related IMEs Tn*6916* and Tn*6917*. Genes are denoted by arrows. Genes, mobile elements and other features are colored based on their functional classification. Shading regions denotes of nucleotide identity ≥ 95%. Numbers in brackets indicate nucleotide positions within the chromosomes of strains FDAARGOS_171 and 918607, respectively. The accession number of Tn*6916* [33] for reference is CP014062

Tn*6916* (a 51.0-kb prototype IME initially identified in *P. monteilii* FDAARGOS_171 [33]) and Tn*6917* were integrated at the same site downstream of the *P. monteilii* chromosomal gene *dinG* (ATP-dependent DNA helicase), and they shared the core backbone markers *attL* (attachment site at the left end), *int* (integrase), and *attR* (attachment site at the right end); in addition, the β-lactam resistance gene *ampC* was considered as a backbone component of these two IMEs because no associated MGEs were identified for this resistance gene (Fig. 4). Tn*6916* thereby had no accessory modules, but *bla*_VIM-2_-carrying In528 (see below) was integrated at a site upstream of *eamA* (transcription regulator) and identified as the sole accessory module in Tn*6917* (Fig. 4).

### Comparison of two related IMEs Tn*6918* and Tn*6919*

**Fig. 5:**
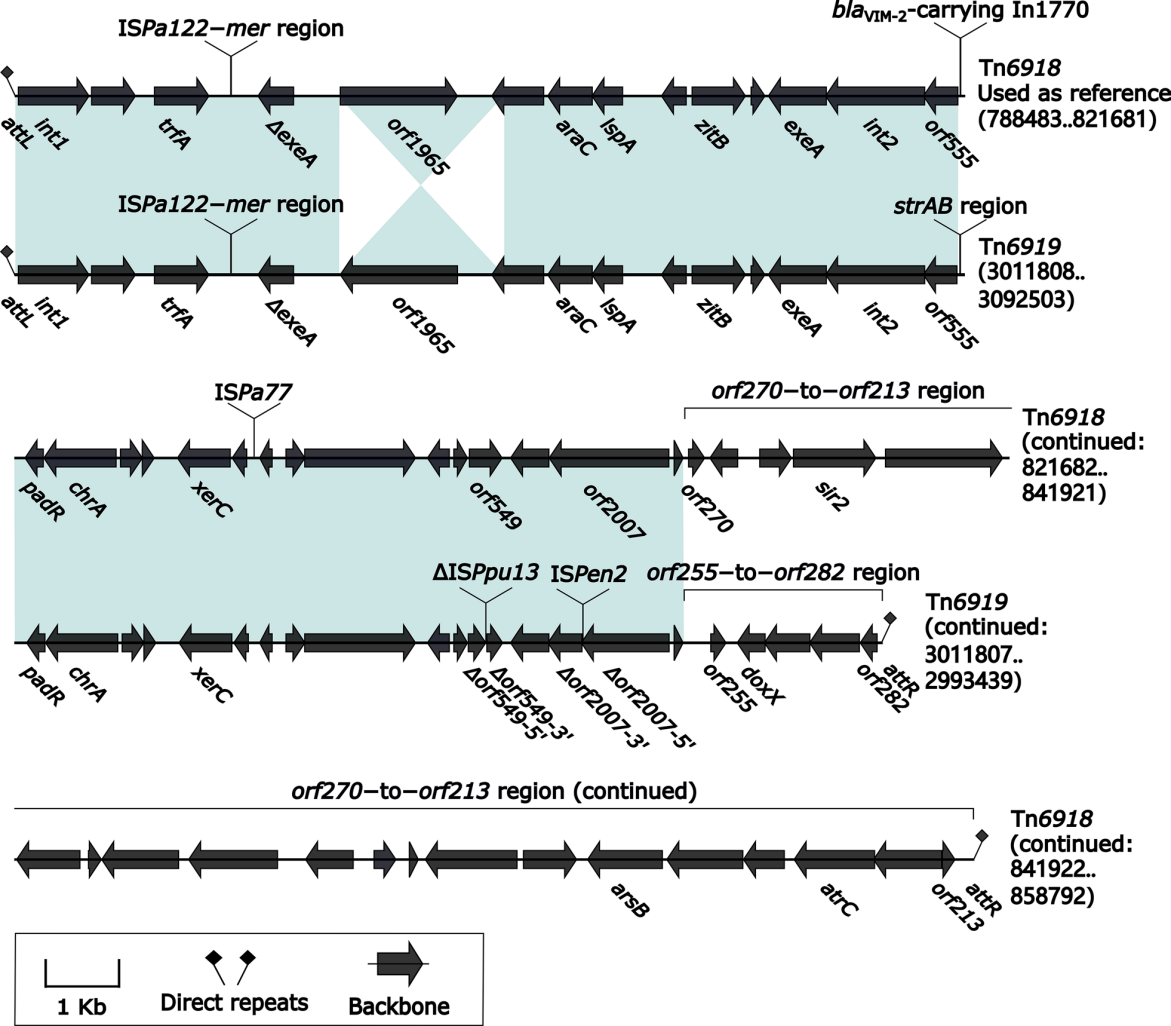
Comparison of the two-related IMEs Tn*6918* and Tn*6919*. Genes are denoted by arrows. Genes, mobile elements and other features are colored based on their functional classification. Shading regions denotes of nucleotide identity ≥ 95%. Numbers in brackets indicate nucleotide positions within the chromosomes of strains 159349 and IEC33019, respectively. The accession number of Tn*6918* for reference is CP045553

Tn*6918* (a 70.49-kb prototype IME initially found in *P. putida* 159,349) and Tn*6919* were integrated at a site downstream of the *P. putida* chromosomal gene tRNA^Thr^, and they shared the core backbone markers *attL*, *int1*, *int2*, and *attR* (Fig. 5). There were at least three major modular differences in the backbone of Tn*6919* relative to Tn*6918*: i) inversion of *orf1965*, ii) interruption of *orf549* and *orf2007* due to insertion of ΔIS*Ppu13* and IS*Pen2*, respectively, and iii) replacement of 3’-termimal 22.8-kb *orf270*–to–*orf213* region by 3.4-kb *orf255*–to–*orf282* region (Fig. 5). The IS*Pa122*–*mer* region was integrated at the same site of the backbones of Tn*6918* and Tn*6919* (Fig. 5), and it was composed of IS*Pa122* and a *mer*-carrying ΔTn*5041*-like element (Additional file 1: Fig. S6). Tn*6918* and Tn*6919* acquired *bla*_VIM-2_-carrying In1770 (see below) and *strAB* region, respectively, which were inserted at the same site upstream of *orf555* (Fig. 5). The *strAB* region contained a cryptic unit transposon Tn*6920*, a *strAB*-carrying unit transposon Tn*6921*, a 3.6-kb Tn*4662a* remnant, and several IS elements (Additional file 1: Fig. S7). Tn*6920* and Tn*6921* had the core transposition *tniABQ*− *res* −*tniR* modules with the highest sequence identities to Tn*5053* [34] and Tn*5058a* [32], respectively, and thus they belonged to the Tn*5053* subfamily of Tn*7* family (Fig. 3).

### Comparison of eight Tn*6417*-related ICEs

**Fig. 6:**
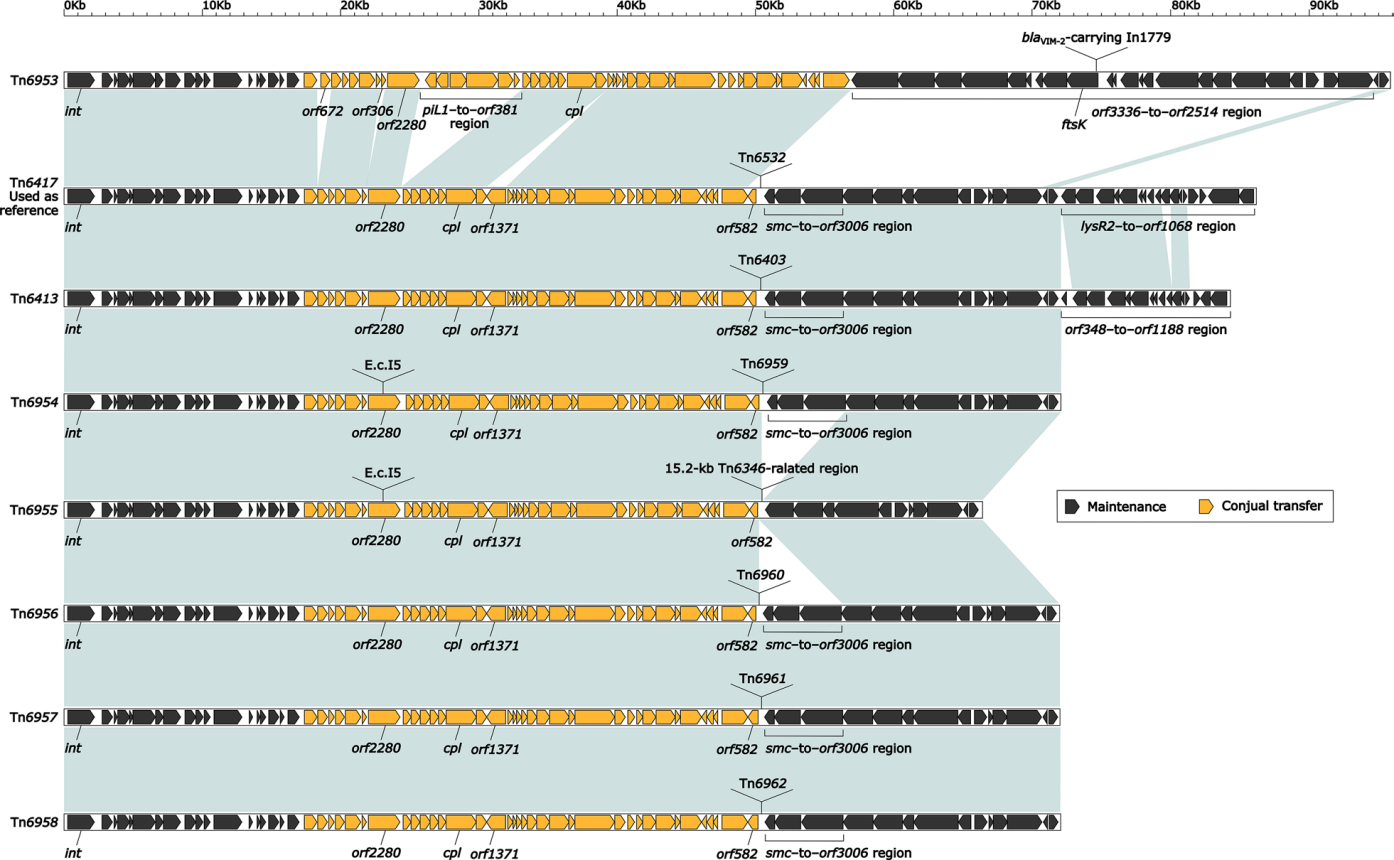
Comparison of Tn*6417* and the seven related ICEs. Genes are denoted by arrows. Genes, mobile elements and other features are colored based on their functional classification. Shading regions denote nucleotide identity ≥ 95%. Accession number of Tn*6417* [7] for reference is EU696790

Tn*6417* was a 108.2-kb reference ICE [7] initially found in *P. aeruginosa* DHS01 [35]. The eight related ICEs Tn*6417*, Tn*6413*, Tn*6953*, Tn*6954* (GZAF3_GI) [36], Tn*6955* (ICE*6440*) [3], Tn*6956* (ICE*6441*) [3], Tn*6957*, and Tn*6958* [37] shared the core backbone markers *attL/attR* (these sequences showed somewhat differences but shared a core motif ‘CCTTCGCCCGCTCCA’), *int*, *cpl* (coupling protein), *rlx* (relaxase), and a F (TivF)-type type IV secretion system gene set (mating pair formation). However, the backbones of these eight ICEs varied in size from 67.8 to 95.1 kb and had at least four major modular differences: (i) presence of unique *orf672*, *orf306*, *piL1*–to–*orf381* region, and *orf3336*–to–*orf2514* region in Tn*6953*; (ii) presence of two highly similar regions *lysR2*–to–*orf1068* and *orf348*–to–*orf1188* in Tn*6417* and Tn*6413*, respectively; (iii) absence of *smc*–to–*orf3006* region from Tn*6955*; and (iv) interruption of *orf2280* owing to insertion of intron E.c.I5 in Tn*6954* and Tn*6955* (Fig. 6). The first six ICEs were integrated into the *P. aeruginosa* or *A. faecalis* chromosomal tRNA^Gly^ gene, while the last two ones into the *P. aeruginosa* chromosomal gene *mhpC* (methyl ester carboxylesterase) and *orf1203* (putative DNA-binding protein), respectively.

**Fig. 7:**
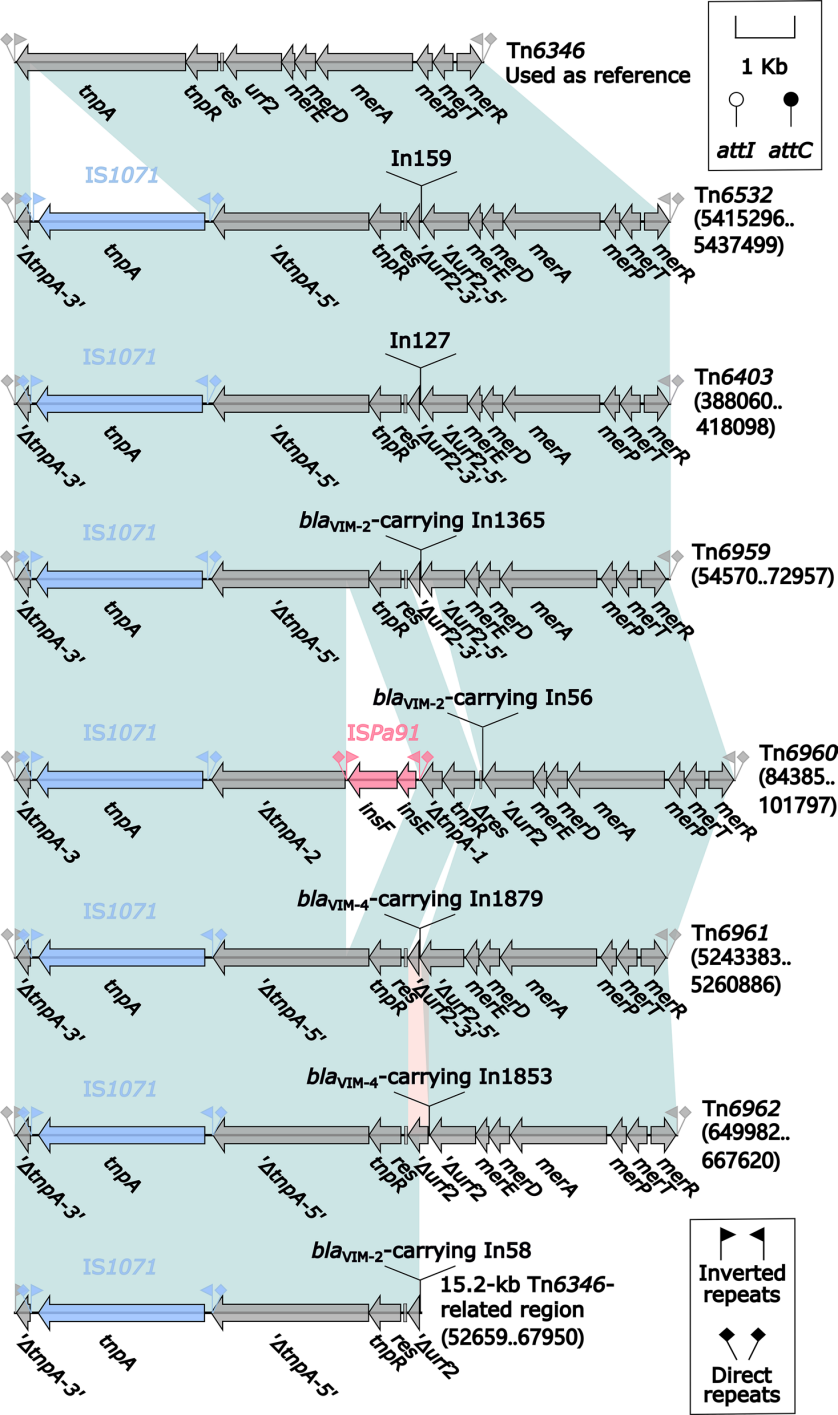
Comparison of Tn*6346* and the seven related elements. Genes are denoted by arrows. Genes, mobile elements and other features are colored based on their functional classification. Shading regions denote nucleotide identity (light blue: ≥95%; and light pink: ˂95% but ≥ 79%). Numbers in brackets indicate nucleotide positions within the chromosomes of strains DHS01, 6762, GZAF3, FFUP_PS_CB5, CCUG 51971, MUB14, and HSV3483, respectively. The accession number of Tn*6346* [38] for reference is EU696790

Each ICE carried a single accessory module, and thus there were the resulting eight different accessory modules *bla*_VIM-2_-carrying In1779 (see below), Tn*6532*, Tn*6403*, Tn*6959*, a 15.2-kb Tn*6346*-related region, Tn*6960*, Tn*6961*, and Tn*6962* from the eight ICEs; the first one was inserted into *ftsk* (cell division protein), while all the other seven ones were integrated at a site upstream of *orf582* and identified as the derivatives of Tn*6346* (Fig. 7). Tn*6346* was a prototype Tn*3*-family unit transposon originally identified in *Achromobacter* spp. AO22 [38] and manifested as a hybrid of the core transposition module *tnpAR*–*res* from Tn*5051* and the *mer* region from Tn*501* (Fig. 7). The seven Tn*6346* derivatives differed from Tn*6346* in two major aspects: (i) insertion of IS*1071* at the same position within *tnpA* in all these seven Tn*6346* derivatives, and furthermore insertion of IS*Pa91* at another position within *tnpA* in only Tn*6960*; and (ii) insertion of different concise class 1 integrons In159, In127, In1365, In56, In1879, In1853, and In58 into *urf2* in these seven Tn*6346* derivatives, sometimes leading to truncation of surrounding regions (Fig. 7). The *bla*_VIM_ genes were found the last five integrons (see below) rather than the first two (Additional file 1: Fig. S8).

### Comparison of eleven *bla*_VIM_-carrying integrons

**Fig. 8:**
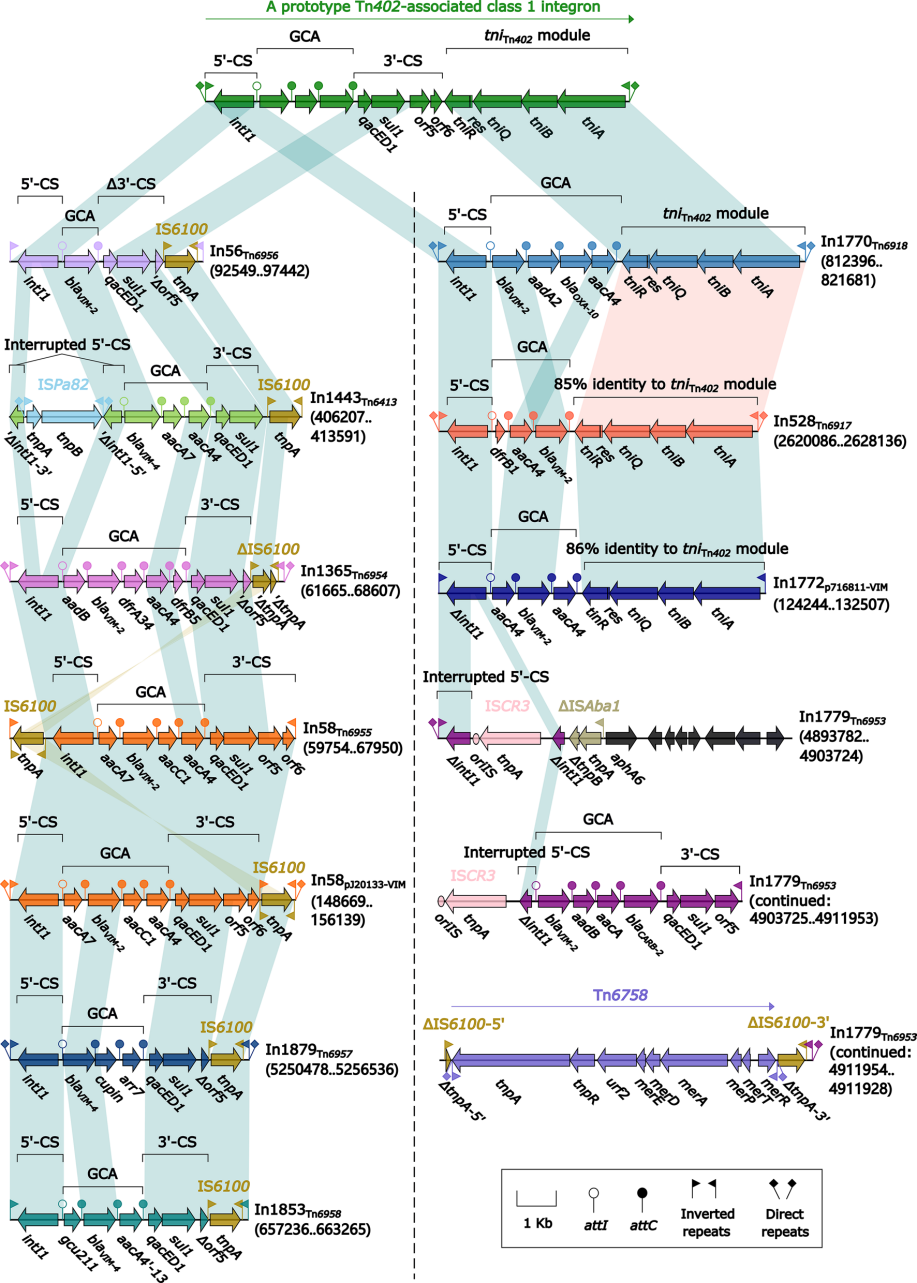
Comparison of the 11 *bla*_VIM_-carrying integrons. Genes are denoted by arrows. Genes, mobile elements and other features are colored based on their functional classification. Shading regions denote of nucleotide identity (light blue and yellow: ≥ 95%; and light pink ˂95% but ≥ 85%). Numbers in brackets indicate nucleotide positions within the corresponding plasmids pJ20133-VIM and p716811-VIM or within the chromosomes of strains FFUP_PS_CB5, 6762, GZAF3, HSV3483, CCUG 51971, MUB14, 159349, 918607, and SE5443

The *bla*_VIM_ genes were found in the two plasmids pJ20133-VIM and p716811-VIM (Table 1) and the nine chromosomal IMEs/ICEs Tn*6917*, Tn*6918*, Tn*6413*, Tn*6953*, Tn*6454*, Tn*6455*, Tn*6456*, Tn*6457* and Tn*6458* (Table 2). The 11 corresponding local *bla*_VIM_ genetic environments manifested as 11 different class 1 concise integrons, and three of them contained *bla*_VIM-4_ while the other nine carried *bla*_VIM-2_ (Fig. 8). A complete class 1 concise integron contained IRi (inverted repeat at the integrase end), 5’-conserved segment (5'-CS: *intI1*–*attI1*), GCA, 3'-CS (*qacED1*–*sul1*–*orf5*–*orf6*), *tni*_Tn*402*_ (Tn*402* core transposition module *tniABQ*–*res*–*tniR*), and IRt (inverted repeat at the *tni* end). Except for In1443_Tn*6413*_ with terminal truncations, all the other tens had the terminal IRi/IRt pairs. Insertions or truncations occurred in 5'-CS or 3'-CS or *tni*_Tn*402*_ of all these 11 integrons, especially including In1779 that showed the longest modular structure because of integration of a *mer*-containing Tn*3*-family prototype unit transposon Tn*6758*. In56_Tn*6596*_ had the shortest GCA that contained only *bla*_VIM-2_, while all the other 10 integrons had the multiple-gene GCAs harboring *bla*_VIM-2_ plus one or more additional antibiotic resistance genes.

### Summary of newly identified or designated MGEs

There were eight newly identified MGEs, including two IMEs Tn*6917* and Tn*6918*, one ICE Tn*6953*, two unit transposons Tn*6735* and Tn*6740*, and three integrons In1770, In1772, and In1779. Additional 13 MGEs (IMEs: Tn*6734*, Tn*6916*, Tn*6919*, and Tn*6957*; unit transposons: Tn*6920*, Tn*6921*, Tn*6959*, Tn*6960*, Tn*6961*, and Tn*6962*; IS elements: IS*Pmo2*, IS*Pmo3*, IS*Pmo5*) were newly designated but with previously determined sequences. The three previously designated ICEs GZAF3_GI, ICE*6440*, and ICE*6441* were renamed as the standard Tn designations Tn*6954*, Tn*6955*, and Tn*6956*, respectively.

### Transferability and antimicrobial susceptibility

pJ20133-VIM, p716811-VIM, and Tn*6953*, which were selected to represent the Inc_pSTY_-1 and Inc_pSTY_-2 plasmids and the Tn*6417*-related ICEs, respectively, could be transferred from the relevant wild-type isolates into ATCC 27853 or PAO1 through conjugation, generating the transconjugants PAO1/pJ20133-VIM, PAO1/p716811-VIM, and ATCC 27853/Tn*6953* respectively. All these wild-type and transconjugant strains had the Ambler class B carbapenemase activity (data not shown) and were resistant to imipenem and meropenem with minimal inhibit concentration (MIC) values ≥ 16 mg/L (Additional file 1: Table S5), owing to production of VIM enzyme.

## Discussion

The present work identifies a novel group of Inc_pSTY_ plasmids, which can be further divided into the three subgroups Inc_pSTY_-1/2/3. A detailed genetic dissection analysis is applied to a collection of 15 *Pseudomonas* MGEs, including three Inc_pSTY_ plasmids, two Tn*6916-*related IMEs, two Tn*6918-*related IMEs, and eight Tn*6417-*related ICEs. All these IMEs/ICEs were located within the bacterial chromosomes. At least 22 genes for resistance to seven different categories of antibiotics and heavy metals are identified within these 15 MGEs (Additional file 1: Table S6). Eleven of these 15 MGEs carry the *bla*_VIM_ genes, which are all located within GCAs of concise class 1 integrons. For 10 of these integrons, the *bla*_VIM_ genes were presented together with other resistance genes, especially including those for resistance to β-lactams, aminoglycosides, trimethoprim, and rifampicin. These *bla*_VIM_-carrying integrons were further integrated into the above plasmids, IMEs and ICEs.

Confirmed by conjugal transfer experiments herein, Inc_pSTY_ plasmids and Tn*6417*-related ICEs have the ability to transfer from one cell to another cell. These two groups MGEs could transfer autonomously by utilizing self-encoded conjugation gene sets [39, 40]. However, the conjugal transfer of Tn*6916*- and Tn*6918*-related IMEs are nonautonomous due to lack of essential conjugation genes encoding Cpl and type IV secretion system, and thereby its intercellular transfer is relied on the help of other conjugative elements [40].

*bla*_VIM_ genes are widely disseminated in *Pseudomonas* [41–43], Enterobacteriaceae [5, 44, 45], *Providencia* [46, 47], *Achromobacter* [48], *Acinetobacter* [49, 50], *Aeromonas* [51], and *Morganella* [52]. Currently, at least 183 different *bla*_VIM_-carrying integrons [19, 53, 54] have been identified, and they either directly integrate into the chromosomes or reside as the inner-components of various MGEs such as plasmids, unit transposons, ICEs and IMEs [3, 55–57]. These results indicate that *bla*_VIM_ genes have evolved to reside diverse MGEs with the intra- or inter-cellular mobility, which will promote the wide dissemination of *bla*_VIM_ genes.

Until now, only seven Inc_pSTY_ plasmids are identified in six *Pseudomonas* isolates and one *Stenotrophomonas rhizophila* isolate, which come from China (n = 5), Japan (n = 1) and Germany (n = 1) (Additional file 1: Table S2). Only six Tn*6916*-related IMEs and two Tn*6918*-related IMEs are detected in eight *Pseudomonas* isolates, two of which are sequenced in this study. The six Tn*6916*-related IMEs come from China (n = 4), USA (n = 1) and Japan (n = 1), while the two Tn*6918*-related IMEs from China (n = 1) and Brazil (n = 1). These observations indicate that these three group MGEs are mainly prevalent in *Pseudomonas* isolates and meanwhile still not widely disseminated in multiple geographic regions. By contrast, Tn*6417*-related ICEs are frequently identified in *Pseudomonas* [7, 58, 59] and also occasionally in *Bordetella*, *Alcaligenes*, *Klebsiella pneumoniae*, *Achromobacter xylosoxidans*, *Morganella morganii*, *Aeromonas caviae*, *Casimicrobium huifangae*, and *Delftia acidovorans*. Tn*6417*-related ICEs have been already disseminated worldwide in a plenty of bacterial species.

## Conclusion

The four groups of *bla*_VIM_-carrying MGEs characterized in this study have a wide range of hosts including *Pseudomonas*, and they are able to transfer across different bacterial species. Integration of *bla*_VIM_ genes into these MGEs contributes to the accumulation and distribution of *bla*_VIM_ genes and enhances the ability of bacteria to survive under selection pressure of carbapenems especially in hospital settings. Moreover, these four groups of MGEs display a high-level diversification in modular structures, which have complex mosaic natures and carry a large number of drug resistance genes (particularly in the MDR regions in these MGEs). This study provides a deeper insight into the genetic diversification and evolution of VIM-encoding MGEs in *Pseudomonas*.

## Supplementary Information

**Additional file 1: Fig S1.** Organization of *paa* region from pSTY. **Fig S2.** Organization of Tn*6734* and Tn*6603a* from pSTY. **Fig S3.** Organization of MDR region from pJ20133-VIM, and comparison with related regions. **Fig S4.** Organization of two Tn*7*-family elements, and comparison with related region. **Fig S5.** Organization of MDR region from p716811-VIM, and comparison with related regions. **Fig S6.** Organization of IS*Pa122* − *mer* region from Tn*6918* and Tn*6919*, and comparison with related region. **Fig S7.** Organization of *strAB* region from Tn*6919*, and comparison with related regions. **Fig S8.** Organization of In159 and In127 from Tn*6532* and Tn*6403* respectively, and comparison with related region. **Table S1.** Major features of the five *Pseudomonas* isolates sequenced in this study. **Table S2.** List of all the seven sequenced Inc_pSTY_ single-replicon plasmids. **Table S3.** Pairwise comparison of *repA*_IncpSTY_ sequences from the seven Inc_pSTY_ plasmids using BLASTN. **Table S4.** Pairwise comparison of backbone sequences from the three Inc_pSTY_ plasmids using BLASTN. **Table S5.** Antimicrobial drug susceptibility profiles. **Table S6.** Resistance genes in the 15 mobile genetic elements (MGEs) analyzed.

## Data Availability

The complete sequences of plasmids pJ20133-VIM and p716811-VIM, and those of the chromosomes of strains SE5443, 918607, and 159349 were submitted to GenBank under accession numbers MN310371, MN310372, CP046405, CP043395, and CP045553 respectively.

## Abbreviations

MGE: Mobile genetic element
ICE: Integrative and conjugative element
IME: Integrative and mobilizable element
GCA: Gene cassette array
Inc: Incompatibility
RAST: Rapid Annotation using Subsystem Technology
BHI: Brain Heart Infusion
CLSI: Clinical and Laboratory Standards Institute
MLST: Multi-locus Sequence Typing
MDR: Multi-drug resistance
IRi: Inverted repeat at the integrase end
CS: Conserved segment
IRt: Inverted repeat at the *tni* end
MIC: Minimal inhibit concentration
