# Supplementary material for: VIM-encoding IncpSTY plasmids and chromosome-borne integrative and mobilizable elements (IMEs) and integrative and conjugative elements (ICEs) in Pseudomonas
Source: Ann Clin Microbiol Antimicrob. 2022 Mar 9;21:10. doi: 10.1186/s12941-022-00502-w (PMC8905914; doi:10.1186/s12941-022-00502-w)
Supplement: Supplementary file 1 — Additional file 1: Fig S1. Organization of paa region from pSTY. Fig S2. Organization of Tn6734 and Tn6603a from pSTY. Fig S3. Organization of MDR region from pJ20133-VIM, and comparison with related regions. Fig S4. Organization of two Tn7-family elements, and comparison with related region. Fig S5. Organization of MDR region from p716811-VIM, and comparison with related regions. Fig S6. Organization of ISPa122 − mer region from Tn6918 and Tn6919, and comparison with related region. Fig S7. Organization of strAB region from Tn6919, and comparison with related regions. Fig S8. Organization of In159 and In127 from Tn6532 and Tn6403 respectively, and comparison with related region. Table S1. Major features of the five Pseudomonas isolates sequenced in this study. Table S2. List of all the seven sequenced IncpSTY single-replicon plasmids. Table S3. Pairwise comparison of repAIncpSTY sequences from the seven IncpSTY plasmids using BLASTN. Table S4. Pairwise comparison of backbone sequences from the three IncpSTY plasmids using BLASTN. Table S5. Antimicrobial drug susceptibility profiles. Table S6. Resistance genes in the 15 mobile genetic elements (MGEs) analyzed. [file 12941_2022_502_MOESM1_ESM.docx]

**Additional file 1 tables and figures**

**Table S1**

Major features of the five *Pseudomonas* isolates sequenced in this study.

| Isolate | Bacterium | Country | Year | Specimen | Host | Sequence type | Source | Accession number |
| --- | --- | --- | --- | --- | --- | --- | --- | --- |
| J20133 | *Pseudomonas monteilii* | China | 2013 | Secreta | Homo sapiens | — | This study | MN310371 |
| 716811 | *Pseudomonas putida* | China | 2015 | Blood | Homo sapiens | ST129 | This study | MN310372 |
| 918607 | *P. monteilii* | China | 2017 | Drainage | Homo sapiens | — | This study | CP043395 |
| 159349 | *P. putida* | China | 2013 | Ascites | Homo sapiens | ST17 | This study | CP045553 |
| SE5443 | *Pseudomonas. aeruginosa* | China | 2012 | Bronchial aspirate | Homo sapiens | ST639 | This study | CP046405 |

**Table S2**

List of all the seven sequenced Inc_pSTY_ single-replicon plasmids.

| **Inc_pSTY_ subtype** | **Plasmid** | **Host bacterium** | **Country** | **Accession number** |
| --- | --- | --- | --- | --- |
| Inc_pSTY_-1 | pSTY | *Pseudomonas taiwanensis* | Germany | CP003962 |
|  | pXWY | *Pseudomonas* spp. | China | CP026333 |
|  | pJ20133-VIM | *P. monteilii* | China | MN310371 |
| Inc_pSTY_-2 | p716811-VIM | *P. putida* | China | MN310372 |
| Inc_pSTY_-3 | pKF715B | *P. putida* | Japan | AP015031 |
|  | pCP031732 | *Stenotrophomonas rhizophila* | China | CP031732 |
|  | pHN39-SIM | *P. aeruginosa* | China | KU254577 |

The *repA* sequences of these seven plasmids were listed below.

>*repA*_pSTY_

ATGGACTCAGAAACCCCCACACCGCGAGCTCCGAGAGCAAAGCGTACAGCCGGAGCGACAACGGATACCAGTCCAGCTAAACGCAAGAGGGCACCCAAGCTCCAGGTCGTTCGTGATGTAGAGCCATCCAAAGCCGTAGAGCTGAAAAAGGCAACCGAAGCCCTCTCGATGCGAAACGTCTCTGCCGCCAGTGACTTCACATTCCTTCAGCGAAAACTCTACAACACCCTCCTTCAATTCGCCCAGCAGCGGCCGAGAGAGGAGATGGTTCATGAGATCCCGATCAAACAGGTCGAGGACAACATTGGACACACCACGTCGAACAGCCGTGATTACCTCAAGAAGGTACTCGTGAGCATGTCTCAGACCCAGGTGGAGTTTGACTACAAGGGTGAAAGCCCTGGGCGCAAGAGCGAATGGGGCATCGCAAACCTGATCGCTGAAGCCTACATCTTGGAAGATGGCCAGACCCTTCGCTTTTCGTTCCCACCGGATCTGAAACGCCGGCTACTCGACCCAGCGATCTTCAACTTGATCGACTTGCGGATGCAGTACCACTTCTCCAGCTTCTCTGCTCTCACTCTGCACGAGATCACGTCTCGCTACCTCGGCTCACCCATGGGAGAGACGTACCGTGCACATTGGTCTGAGTGGAGCGTTGTGCTGTCAGGATCTGCTACGCCACATGCCGAATTTCGCGACTTCAACAAAATGCTGGGTCGCGCAATCGACCAGGTCAACAGCATTGAACGCAGATTCCGCATCTCACCGCACGTCACAAAGCTAAACCGGAAGATGGACAAGCTTTGGTTCAAGCTGGAGACACTGATTCAGCCTGGCCTAGACCTTGGACCATCACCAGAGCTGGTTAGCCAGGACGTGTCAAAGCGCCTTAAAGCACTGTCCTTGAGTCAAAAAGATATCGATGAGCTTGGCATGACTCACGATGAAGAGTACCTGCTTGCGCAAGCTGACTACACCGAGGCACAGATGCGCAAGGAAGGGGCTAACGTTGCAAGTCCAGCGGCATATTTCAAGGCTGCAGTAGCCAACAACTACGCCAAGGCGCCAACTCAACAGAAGGCCGCGGAACCAGGCAGGGGCAAAAAGCCAGCCGCCTCTAAAACCGGTGAGAAGTCGAAGCCGGCGCAGGCGCCATCGCAAGCGCCGAAAGCAGCACCATCGAACCAAATGGCAGACCTGCTGGAGCAGTGGGGAGCAGCTCAGCGTGAAGCTATTCGCGCCCAGTTCATGGAACTGTCGGACGAGCAGAAGAAAGAGCTTGCGGAGAAATATGAGACCGAGCTGCGCAAAGACGATCTGGCCTACTCGCAATACCGGTCGAAGGGTTTGAACACGATGGTGATCAATTGCTTGGTAGCGATCCAGTTCCAGGAGCGTTTCCCTGAGACCCCAAGCTCGGAAACACTCCTGCAATTCTTGCTGGGTGGAGCGAAGATCTAA

>*repA*_pXWY_

ATGGACTCAGAAACCCCCACACCGCGAGCTCCGAGAGCAAAGCGTACAGCCGGAGCGACAACGGATACCAGTCCAGCGAAACGCAAGAGGGCACCCAAGCTCCAGGTCGTTCGTGATGTAGAGCCATCCAAAGCCGTAGAGCTGAAAAAGGCAACCGAAGCCCTCTCGATGCGAAACGTCTCTGCCGCCAGTGACTTCACGTTTCTTCAGCGAAAACTCTACAACACCCTCCTTCAATTCGCCCAGCAGCGGCCGAGAGAGGAGATGGTTCATGAGATCCCGATCAAACAGGTCGAGGACAACATTGGACACACCACATCGAACAGCCGTGATTACCTCAAGAAGGTACTCGTGAGCATGTCTCAGACACAGGTGGAGTTTGACTACAAGGGTGAAAGCCCGGGGCGGAAGAGCGAATGGGGCATCGCAAACCTGATCGCTGAAGCCTACATCTTGGAAGATGGCCAGACCCTTCGCTTTTCGTTCCCACCGGATCTGAAACGCCGGCTACTCGACCCAGCGATCTTCAACTTGATCGACTTGCGGATGCAGTACCACTTCTCCAGCTTCTCTGCTCTCACTCTGCACGAGATCACGTCTCGATACCTGGGCTCACCTATGGGAGAGACGTACCGTGCACATTGGTCTGATTGGAGCGTTGTGCTGTCCGGATCTGCTACGCCACATGCCGAATTTCGCGACTTCAACAAAATGCTGGGTCGCGCAATCGACCAGGTCAACAGCATTGAACGCAGATTCCGCATCACACCGCACGTCACTAAGCTAAATCGGAAGATGGACAAGCTTTGGTTCAAGCTGGAGACACTGATTCAGCCTGGCCTAGACCTTGGACCATCACCAGAGCTGGTCAGCCAGGACGTGTCAAAGCGCCTTAAAGCACTGTCGTTGAGTCAAAAGGATATTGATGAGCTTGGCATGACTCACGATGAAGAGTACCTGCTTGCGCAAGCTGACTACACCGAGGCACAGATGCGCAAGGAAGGGGCTAACGTTGCAAGTCCAGCGGCATATTTCAAGGCTGCAGTAGCCAACAACTACGCCAAGGCGCCAACTCAACAGAAGGCCGCGGAACCAGGCAGAGGCAAAAAGCCAGCCGCCTCTAAAAGCGGTGAGAAGTCGAAGCCGGCGCCATCGCAAGCGCCGAAAGCAGCACCATCGAACCAAATGGCAGACCTACTGGAGCAGTGGGGAGCAGCTCAGCGTGAAGCTATCCGCGCCCAGTTCATGGAACTGTCGGATGAGCAGAAGAAAGAGCTTGCGGAGAAATATGAGACCGAGCTGCTCAAAGACGATCTGGCCTACTCGCAATACCGGTCGAAGGGTTTGAACACGATGGTGATCAATTGCTTGGTAGCGATCCAGTTCCAGGAGCGTTTCCCTGAGCCCCCAAGCTCGGAAACACTCCTGCAATTCTTGCTAGGTGGAGCGAAGATCTAA

>*repA*_pJ20133-VIM_

ATGGACTCAGAAACCCCCACACCGCGAGCTCGGAGAGCAAAGCGTACAGCCGGAGCGACAACGGATACCAGTCCAGCGAAACGCAAGAGAGCACCCAAGCTCCAGGTCGTTCGTGATGTAGAACCCTCCAAAGCCGTCGAGCTGAGAAAGGCAACCGAAGCCCTTTCGATGCGAAACGTCTCTGCCGCCAGTGACTTCACATTCCTTCAGCGAAAGCTCTATAACACCCTCCTGCAATTCGCCCAGCAGCGGCCGAGAGATGAGATGGTTCATGAGATCCCGATCAAGCAGGTCGAGGACAACATTGGACACACCACCTCGAACAGCCGTGATTACCTCAAGAAGGTACTCGTGAGTATGTCTCAGACACAGGTGGAGTTTGACTACAAGGGTGAAAGCCCTGGCCGCAAGAGCGAGTGGGGCATCGCAAACCTGATCGCTGAAGCCTACATCTTGGAAGATGGCCAGACACTTCGCTTCTCGTTCCCGCCTGATCTGAAACGCCGGCTACTCGACCCTGCGATCTTCAACTTGATCGACTTGCGTATGCAGTACCACTTCTCCAGCTTTTCTGCTCTCACTCTCCACGAAATCACGTCGCGCTACCTCGGCTCACCCATGGGGGAGACGTACCGTGCACATTGGTCTGAATGGAGCGTTGTGCTGTCAGGATCTGCTACGCCACATGCCGAATTTCGCGACTTCAACAAAATGCTGGGTCGCGCAATCGACCAGGTCAACAGCATTGAACGCAGATTCCGCATCACGCCGCACGTGACAAAGCTAAACCGGAAGATGGACAAGCTTTGGTTCAAGCTGGAGACACTGATTCAGCCTGGCCTAGACCTTGGACCATCACCAGAGCTGGTTAGCCAGGACGTGTCAAAGCGCCTCAAAGCACTGTCCTTGAGTCAAAAGGATATCGATGAGCTTGGCATGACTCACGACGAAGAGTACCTGCTTGCGCAAGCTGACTACACCGAGGCACAGATGCGCAAGGAAGGGGCTAACGTTGCAAGTCCTGCGGCATATTTCAAGGCTGCAGTAGCCAACAACTACGCCAAGGCTCCAACTCAACAGAAGGCCGCGGAACCAGGCAAAGGCAAAAAGCCAGCCGCCTCTAAAACCGGTGAGAAGTCAAAGCCGGCACCATCGCAAGCGCCGAAAGCAGCACCATCGAACCAAATGGCAGACCTGTTGGAGCAGTGGGGAGCAGCTCAGCGTGAAGCTATCAGGGCCCAGTTCATGGAACTGTCGGACGAGCAGAAGAAAGAGCTTGCGGAGAAATATGAGACCGAGCTGCGCAAAGACGATCTGGCCTACTCGCAATACCGGTCGAAGGGTTTGAACACGATGGTGATCAATTGCTTGGTAGCGATCCAGTTCCAGGAGCGTTTCCCTGAGGCCCCAAGCTCGGAAACACTCCTGCAATTCTTGCTGGGTGGAGCGAAGATCTAA

>*repA*_p716811-VIM_

ATGGACTCAGAAACCCCCACACCGCGAGCTACGCGACAAAAGCGTACAGCCGGAGCAACAACGGATACTAGTCCAGCAAAACGCAAGAGAGCACCCAAGCTCCAAGTCGTTCGTGATGTTGAACCATCCAAAGCCGTCGAGCTGAAAAAGGCCACCGAAGCCCTTTCGATGCGAAACGTCTCTGCCGCCAGTGACTTCACATTCCTTCAGCGAAAGCTCTATAACACCCTCCTTCAATTCGCTCAGCAGCGGCCGAGGGAGGAGATGGTTCACGAGATCCCGATCAAGCAGGTCGAGGACAACATTGGGCACACCACCTCGAACAGCCGTGATTACCTCAAAAAGGTACTCGTGAGCATGTCTCAGACGCAGGTTGAATTTGACTACAAGGGTGAAAGCCCTGGGCGGAAGAGCGAATGGGGCATCGCAAACCTGATCGCTGAAGCCTACATCTTGGAAGATGGGCAGACCCTCCGCTTTTCGTTCCCGCCTGATCTGAAACGCCGACTACTGGACCCCGCGATCTTCAACCTGATCGACTTGCGGATGCAGTACCATTTCTCCAGCTTTTCTGCGCTCACTCTCCACGAGATCACATCTCGCTACCTCGGTTCACCCATGGGGGAGACCTACCGTGCACATTGGTCCGAGTGGAGCGTTGTGCTGTCTGGATCTGCTACACCACATGCCGAGTTTCGCGACTTCAACAAAATGCTGGGTCGCGCAATCGACCAGGTCAACAGCATTGAACGGAGATTTCGCATCACGCCGCACGTCACCAAGCTAAACCGGAAGATGGACAAGCTTTGGTTCAAGCTGGAGACCCTGATTCAGCCTGGCTTAGACCTTGGACCATCACCAGAGCTGGTTAGCCAGGATGTGTCAAAGCGCCTTAAAGCTCTGTCCTTGAGTCAAAAGGATATCGCTGAGCTTGGCATGACCCATGACGAAGAGTATCTGCTAGCGCAAGCTGACTACACCGAGGCACAGATGCGTAAAGAGGGGGCTAACGTTGCAAGTCCAGCGGCATATTTCAAGGCCGCGGTAGCCAACAACTACGCCAAGGCGCCAACTCAACAGAAAGCCGCGGAGACAGGCAAAGGCAAAAAGCCGGCCGCCTCAAAATCAGGTGATAAGGCAAAGCCTGCACCAGCGCCTTCGCAAGCGCCAAAAGCAGCACCGTCGAACCAAATGGCAGATCTGTTGGAACAGTGGGGTGCTGCTCAGCGTGAAGCAATCCGCGCCCAGTTCATGGTGTTGTCGGATGAGCAGAAGAAAGAGCTCGCGGAGAAATATGAGACCGAGCTACGCAAAGATGATCTGGCCTACTCGCAATACCGGTCGAAGGGTTTGAACACCATGGTGATCAATTGCTTGGTAGCGATCCAGTTCCAGGAGCGTTTCCCTGAGACCCCAAGCTCGGAAACACTCCTGCAATTCTTGCTGGGTAGTGCGAAGATCTAA

>*repA*_pKF715B_

ATGGACTCAGAAACCCCCACACCGAGAGCTCCGCGAGCAAAGCGTACAACCGGAGCAACAACGGAAACTAGTCCAGCGAAACGCAAGAGAGCACCAAAGCTCCAGGTCGTTCGAGATGTCGAACCATCCAAAGCCGTTGAGCTGAGGAAGGCGACCGAAGCCCTGTCAATGCGAAACGTCTCCGCCACTAGTGACTTCACCTTCCTACAGCGAAAACTCTACAACACACTCCTGCAGTTCGCCCAGCAACGGCCACGGGAGGAGATGGTTCACGAGATCCCGATTCGAGACGTCGAAGACAACATTGGACATACCACGTCCAATAGCCGCGACTACCTCAAAAAGGTGCTCGTGAGCATGTCGCAGACGCAGGTTGAGTTTGACTACAAGGGTGAGAGCCCTGGCCGAAAAAGCGAGTGGGGTATCGCAAACCTAATTGCTGAAGCCTACATCCTTGAGGATGGGCAAACCCTGCGCTTCTCGTTCCCACCAGATCTGAAACGCCGGCTGCTCGACCCAGCGATCTTCAATTTGATCGATCTGCGGATGCAGTACCACTTTTCAAGCTTTTCAGCGCTTACGCTCCACGAGATCACGTCACGCTATTTTGGCTCACCCATGGGTGAGACCTACCGTGCGCACTGGTCTGAGTGGAGCGTTGTGTTGTCTGGATCAGCCACTCCGCACGCCGAGTTTCGCGATTTCAACAAGATGCTTGGTCGTGCTATCGACCAGGTCAACAGCATCGAGCGAAGATTCCGCATCACTCCGCACGTGACCAAGGCCAGCCGGAAGATGGATAAGCTCTGGTTCAAATTGGAGACCTTGGTTCAACCAGGCTTAGACCTGGGACCATCCCCCGAGCTGGTTAGCCAGGACGTATCCAAGCGCCTGAAGGCACTGTCCTTGAGCAAAAAGGACATCGATGAGCTCGGGATGACTCATGATGAAGAATACCTCTTAGCTCAAGCTGACTACACAGAAGCGCAAATGCGCAAGGAGGGGGCCAACGTTGCAAGTCCCGCGGCATATTTCAAGGCTGCGGTCGCCAACAACTACGCCAAGGCGCCAACCCAGAAGAAGGGAGCGGAAGCTGCCAAATCCCAGAAGCCAGCGGCTAACGCTACTAAGCCAGCTGCGAAAGCAAAGCCAGCGCCGAAAGCAGCACCAACGAATCAAATGGCTGACCTTTTGGAGCAGTGGGGAGCTGCTCAACGCGAGACTATCCGTGGCGAGTTCCAGGGGTTGTCGGATGAAGAAAAGAACGAGCTGGCTGAGAAATATGACGCCGAGCTGAGGAAGGATGACTTCGCTTACGGGCAGTACCGGACCAAAGGATTGACTCCTATGGTGACCAATTGCTTGGTGGCAATCTTGTTCCAGGAACGCTTCCCTGAGACTCCGACCCCGGAAACGCTCCTTCAATTCTTGCTTAGTGGCGGCAAGATCT

>*repA*_pCP031732_

ATGGACTCAGAAACCCCCACACCGAGAGCTCCGCGAGCAAAGCGTACAACCGGAGCAACAACGGAAACTAGTCCAGCGAGACGCAAGAGAGCACCAAAGCTCCAGGTCGTTCGAGATGTCGAACCATCCAAAGCCGTTGAGCTGAGGAAGGCTACCGAAGCCCTGTCAATGCGAAACGTCTCCGCCACTAGTGACTTCACCTTCCTACAGCGAAAACTCTACAACACGCTCCTGCAGTTCGCCCAGCAACGGCCACGGGAGGAGATGGTTCACGAGATCCCGATTCGAGACGTCGAAGACAACATTGGACACACCACGTCCAATAGCCGCGACTACCTCAAAAAGGTGCTCGTGAGCATGTCGCAGACGCAGGTTGAGTTTGACTACAAAGGTGAGAGCCCTGGCCGAAAAAGCGAGTGGGGTATCGCAAACCTAATTGCTGAAGCCTACATCCTTGAGGATGGGCAAACCCTGCGCTTCTCGTTCCCACCAGATCTGAAACGCCGGCTGCTCGACCCAGCGATCTTCAATTTGATCGATCTGCGGATGCAGTACCACTTTTCAAGCTTTTCAGCGCTCACGCTCCACGAGATCACGTCACGCTATTTTGGTTCACCCATGGGTGAGACCTACCGTGCGCACTGGTCTGAGTGGAGCGTTGTGTTATCTGGATCAGCCACTCCGCACGCCGAGTTTCGCGATTTCAACAAGATGCTTGGTCGTGCTATCGACCAGGTCAACAGCATCGAGCGAAGATTCCGCATCACTCCGCACGTGACCAAGGCCAACCGGAAGATGGATAAACTCTGGTTCAAATTGGAGACCTTGGTTCAACCAGGCTTAGACTTGGGACCATCCCCGGAGCTGGTTAGCCAGGACGTATCCAAGCGCCTGAAAGCACTGTCCTTGAGCAAAAAGGATATCGATGAGCTCGGGATGACTCATGATGAAGAATACCTCCTAGCTCAAGCTGACTACACAGAAGCGCAAATGCGCAAGGAAGGGGCCAACGTTGCAAGTCCCGCGGCATATTTCAAGGCTGCGGTCGCCAACAACTACGCCAAGGCGCCAACCCAACAGAAGGGAGTGGAATCTGGCAAATCCCAGAAGCCATCGGCTAACGCTACTAAGCCAGCTGCGAAACCAAAGCCAGCGCCGAAAGCAGCACCAACGAATCAAATGGCAGACCTTTTGGAGCAGTGGGGAGCTGCTCAACGCGAGACTATCCGTGGCGAGTTCCAGGGGTTGTCGGATGAAGAAAAGAACGAGCTGGCAGAGAAGTATGACGCCGAGCTGAGGAAGGATGACTTCGCTTACGGGCAGTACCGAACCAAAGGATTGACCCCTATGGTGACCAATTGCTTGGTGGCAATCCTGTTCCAGGAACGCTTCCCTGAGACTCCGACCCCGGAAACGCTCCTTCAATTCTTGCTTAGTGCCGGCAAGATCT

>*repA*_pHN39-SIM_

ATGGACTCAGAAACCCCCACACCGAGAGCTCCGCGAGCAAAGCGTACAACCGGAGCAACAACGGAAACTAGTCCAGCGAGACGCAAGAGAGCACCAAAGCTCCAGGTCGTTCGAGATGTCGAACCATCCAAAGCCGTTGAGCTGAGGAAGGCTACCGAAGCCCTGTCAATGCGAAACGTCTCCGCCACTAGTGACTTCACCTTCCTACAGCGAAAACTCTACAACACGCTCCTGCAGTTCGCCCAGCAACGGCCACGGGAGGAGATGGTTCACGAGATCCCGATTCGAGACGTCGAAGACAACATTGGACACACCACGTCCAATAGCCGCGACTACCTCAAAAAGGTGCTCGTGAGCATGTCGCAGACGCAGGTTGAGTTTGACTACAAAGGTGAGAGCCCTGGCCGAAAAAGCGAGTGGGGTATCGCAAACCTAATTGCTGAAGCCTACATCCTTGAGGATGGGCAAACCCTGCGCTTCTCGTTCCCACCAGATCTGAAACGCCGGCTGCTCGACCCAGCGATCTTCAATTTGATCGATCTGCGGATGCAGTACCACTTTTCAAGCTTTTCAGCGCTCACGCTCCACGAGATCACGTCACGCTATTTTGGTTCACCCATGGGTGAGACCTACCGTGCGCACTGGTCTGAGTGGAGCGTTGTGTTATCTGGATCAGCCACTCCGCACGCCGAGTTTCGCGATTTCAACAAGATGCTTGGTCGTGCTATCGACCAGGTCAACAGCATCGAGCGAAGATTCCGCATCACTCCGCACGTGACCAAGGCCAACCGGAAGATGGATAAACTCTGGTTCAAATTGGAGACCTTGGTTCAACCAGGCTTAGACTTGGGACCATCCCCGGAGCTGGTTAGCCAGGACGTATCCAAGCGCCTGAAAGCACTGTCCTTGAGCAAAAAGGATATCGATGAGCTCGGGATGACTCATGATGAAGAATACCTCCTAGCTCAAGCTGACTACACAGAAGCGCAAATGCGCAAGGAAGGGGCCAACGTTGCAAGTCCCGCGGCATATTTCAAGGCTGCGGTCGCCAACAACTACGCCAAGGCGCCAACCCAACAGAAGGGAGTGGAATCTGGCAAATCCCAGAAGCCATCGGCTAACGCTACTAAGCCAGCTGCGAAACCAAAGCCAGCGCCGAAAGCAGCACCAACGAATCAAATGGCAGACCTTTTGGAGCAGTGGGGAGCTGCTCAACGCGAGACTATCCGTGGCGAGTTCCAGGGGTTGTCGGATGAAGAAAAGAACGAGCTGGCAGAGAAGTATGACGCCGAGCTGAGGAAGGATGACTTCGCTTACGGGCAGTACCGAACCAAAGGATTGACCCCTATGGTGACCAATTGCTTGGTGGCAATCCTGTTCCAGGAACGCTTCCCTGAGACTCCGACCCCGGAAACGCTCCTTCAATTCTTGCTTAGTGCCGGCAAGATCTGA

**Table S3**

Pairwise comparison of *repA*_IncpSTY_ sequences from the seven Inc_pSTY_ plasmids using BLASTN.

| (Coverage+  Identity) | pSTY | pXWY | pJ20133-VIM | p716811-VIM | pKF715B | pCP031732 | pHN39-SIM |
| --- | --- | --- | --- | --- | --- | --- | --- |
| pSTY |  | 100%+98% | 100%+96% | 100%+93% | 99%+83% | 99%+83% | 99%+83% |
| pXWY | 100%+98% |  | 100%+96% | 100%+92% | 99%+82% | 99%+83% | 99%+83% |
| pJ20133-VIM | 100%+96% | 100%+96% |  | 100%+93% | 99%+83% | 99%+83% | 99%+83% |
| p716811-VIM | 100%+93% | 100%+92% | 100%+93% |  | 99%+82% | 99%+83% | 99%+83% |
| pKF715B | 100%+83% | 100%+82% | 100%+83% | 100%+82% |  | 100%+98% | 100%+98% |
| pCP031732 | 100%+83% | 100%+83% | 100%+83% | 99%+83% | 100%+98% |  | 100%+100% |
| pHN39-SIM | 99%+82% | 99%+82% | 99%+83% | 98%+82% | 99%+98% | 99%+100% |  |

**Table S4**

Pairwise comparison of backbone sequences from the three Inc_pSTY_ plasmids using BLASTN.

| (Coverage+  Identity) | pSTY | pJ20133-VIM | p716811-VIM |
| --- | --- | --- | --- |
| pSTY |  | 74%+96% | 66%+90% |
| pJ20133-VIM | 93%+96% |  | 85%+91% |
| p716811-VIM | 67%+90% | 68%+91% |  |

**Table S5. Antimicrobial drug susceptibility profiles**

| Antibiotics | Minimum inhibitory concentration (mg/L)/antimicrobial susceptibility | | | | | | | | |
| --- | --- | --- | --- | --- | --- | --- | --- | --- | --- |
|  | **J20133** | **PAO1/**  **pJ20133-VIM** | **716811** | **PAO1/**  **p716811-VIM** | **SE5443** | **ATCC27853/**  **Tn*6953*** | **PAO1** | **ATCC27853** |  |
| Piperacillin | 32/I | ≤4/S | 32/I | 16/S | ≥64/R | ≥128/R | ≤4/S | 8/S |  |
| Ceftazidime | ≥64/R | ≥64/R | ≥64/R | ≥64/R | ≥16/R | ≥128/R | 2/S | 2/S |  |
| Imipenem | ≥16/R | ≥16/R | ≥16/R | ≥16/R | ≥16/R | ≥16/R | 2/S | ≤0.25/S |  |
| Meropenem | ≥16/R | ≥16/R | ≥16/R | ≥16/R | ≥16/R | ≥16/R | 1/S | ≤1/S |  |
| Aztreonam | ≥64/R | 4/S | ≥64/R | ≤1/S | ≥16/R | 32/R | 4/S | 4/S |  |
| Amikacin | ≤2/S | ≤2/S | ≤2/S | 8/S | ≥32/R | ≥64/R | ≤2/S | 4/S |  |
| Gentamicin | 4/S | ≤1/S | 8/I | ≥16/R | ≥8/R | ≥16/R | ≤1/S | ≤1/S |  |
| Ciprofloxacin | 2/I | 0.5/S | 2/I | ≥4/R | ≥2/R | ≥4/R | 0.5/S | 2/I |  |
| Levofloxacin | 4/I | 0.5/S | 4/I | ≥8/R | ≥8/R | ≥8/R | 0.5/S | 2/I |  |

S=sensitive; R=resistant; I= intermediately resistant

**Table S6**

Resistance genes in the 15 mobile genetic elements (MGEs) analyzed.

| **MGEs** | **Resistance marker** | **Resistance phenotype** | **Nucleotide position** | **Subregion located** |  |
| --- | --- | --- | --- | --- | --- |
| pSTY | *mer* locus | Mercuric resistance | 85827..89480 | *paa* region |  |
| pJ20133-VIM | *bla*_VIM-2_ | β-lactam resistance | 153361..154161 | MDR region |  |
|  | *aacA7* | Aminoglycoside resistance | 154314..154772 |  |  |
|  | *aacC1* | Aminoglycoside resistance | 152803..153267 |  |  |
|  | *aacA4* | Aminoglycoside resistance | 152128..152682 |  |  |
|  | *qacED1* | Quaternary ammonium compound resistance | 151612..151959 |  |  |
|  | *sul1* | Sulphonamide resistance | 150779..151618 |  |  |
|  | *mer* locus | Mercuric resistance | 198206..201478  207555..211208 |  |  |
| p716811-VIM | *bla*_VIM-2_ | β-lactam resistance | 126255..127055 | MDR region |  |
|  | *aacA4* | Aminoglycoside resistance | 125610..126164 |  |  |
|  | *qnrVC1* | Quinolone resistance | 123146..123841 |  |  |
|  | *mer* locus | Mercuric resistance | 108777..112739 |  |  |
| Tn*6916* | *ampC* | β-lactam resistance | 4739579..4740721 | Backbone |  |
| Tn*6917* | *bla*_VIM-2_ | β-lactam resistance | 2624899..2625699 | In528 |  |
|  | *aacA4* | Aminoglycoside resistance | 2625790..2626344 |  |  |
|  | *dfrB1* | Trimethoprim resistance | 2626473..2626709 |  |  |
|  | *ampC* | β-lactam resistance | 2630940..2632082 |  |  |
| Tn*6918* | *bla*_VIM-2_ | β-lactam resistance | 813783..814583 | In1770 |  |
|  | *bla*_OXA-10_ | β-lactam resistance | 815497..816297 |  |  |
|  | *aadA2* | Aminoglycoside resistance | 814683..815462 |  |  |
|  | *aacA4* | Aminoglycoside resistance | 816290..816883 |  |  |
|  | *mer* locus | Mercuric resistance | 796448..799745 | IS*Pa122*-*mer* region | |
| Tn*6919* | *strA* | Aminoglycoside resistance | 3063156..3063959 | *strAB* region |  |
|  | *strB* | Aminoglycoside resistance | 3062320..3063156 |  |  |
|  | *mer* locus | Mercuric resistance | 3081175..3084472 |  |  |
| Tn*6417* | *aadB* | Aminoglycoside resistance | 5423757..5424290 | Tn*6532* |  |
|  | *qacED1* | Quaternary ammonium compound resistance | 5424447..5424794 |  |  |
|  | *sul1* | Sulphonamide resistance | 5424788..5425627 |  |  |
|  | *mer* locus | Mercuric resistance | 5433944..5437461 |  |  |
| Tn*6413* | *bla*_VIM-4_ | β-lactam resistance | 409196..409996 | Tn*6403* |  |
|  | *aadA2* | Aminoglycoside resistance | 398558..399337 |  |  |
|  | *aacA4* | Aminoglycoside resistance | 410731..411249 |  |  |
|  | *aacA7* | Aminoglycoside resistance | 410090..410548 |  |  |
|  | *msr*(E) | Macrolide, Lincosamide and Streptogramin B resistance | 402951..404426 |  |  |
|  | *mph*(E) | Macrolide resistance | 404482..405366 |  |  |
|  | *qacED1* | Quaternary ammonium compound resistance | 411418..411765 |  |  |
|  | *sul1* | Sulphonamide resistance | 411759..412598 |  |  |
|  | *mer* locus | Mercuric resistance | 414543..418060 |  |  |
| Tn*6953* | *bla*_VIM-2_ | β-lactam resistance | 4906548..4907348 | In1779 |  |
|  | *bla*_CARB-2_ | β-lactam resistance | 4908715..4909581 |  |  |
|  | *aadB* | Aminoglycoside resistance | 4907436..4907969 |  |  |
|  | *aacA* | Aminoglycoside resistance | 4908043..4908597 |  |  |
|  | *aphA6* | Aminoglycoside resistance | 4898307..4899086 |  |  |
|  | *qacED1* | Quaternary ammonium compound resistance | 4909798..4910145 |  |  |
|  | *sul1* | Sulphonamide resistance | 4910139..4910978 |  |  |
|  | *mer* locus | Mercuric resistance | 4916887..4920407 |  |  |
| Tn*6954* | *bla*_VIM-2_ | β-lactam resistance | 63643..64443 | Tn6*959* |  |
|  | *aadB* | Aminoglycoside resistance | 63031..63564 |  |  |
|  | *aacA4* | Aminoglycoside resistance | 65115..65669 |  |  |
|  | *dfrA34* | Trimethoprim resistance | 64556..65032 |  |  |
|  | *dfrB5* | Trimethoprim resistance | 65800..66036 |  |  |
|  | *qacED1* | Quaternary ammonium compound resistance | 66249..66596 |  |  |
|  | *sul1* | Sulphonamide resistance | 66593..67429 |  |  |
|  | *mer* locus | Mercuric resistance | 69402.. 72919 |  |  |
| Tn*6955* | *bla*_VIM-2_ | β-lactam resistance | 62615..63415 | 15.2-kb In58-carrying Tn*6346*-related region |  |
|  | *aacA7* | Aminoglycoside resistance | 62004..62462 |  |  |
|  | *aacC1* | Aminoglycoside resistance | 63509..63973 |  |  |
|  | *aacA4* | Aminoglycoside resistance | 64094..64648 |  |  |
|  | *qacED1* | Quaternary ammonium compound resistance | 64817..65164 |  |  |
|  | *sul1* | Sulphonamide resistance | 65158..65997 |  |  |
| Tn*6956* | *bla*_VIM-2_ | β-lactam resistance | 93931..94731 | Tn*6960* |  |
|  | *qacED1* | Quaternary ammonium compound resistance | 94918..95265 |  |  |
|  | *sul1* | Sulphonamide resistance | 95259..96098 |  |  |
|  | *mer* locus | Mercuric resistance | 98242..101759 |  |  |
| Tn*6957* | *bla*_VIM-4_ | β-lactam resistance | 5251865..5252665 | Tn*6961* |  |
|  | *arr7* | Rifampicin resistance | 5253332..5253784 |  |  |
|  | *qacED1* | Quaternary ammonium compound resistance | 5254007..5254354 |  |  |
|  | *sul1* | Sulphonamide resistance | 5254348..5255187 |  |  |
|  | *mer* locus | Mercuric resistance | 5257331..5260848 |  |  |
| Tn*6958* | *bla*_VIM-4_ | β-lactam resistance | 659107..659907 | Tn*6962* |  |
|  | *aacA4'-13* | Aminoglycoside resistance | 660015..660572 |  |  |
|  | *qacED1* | Quaternary ammonium compound resistance | 660741..661088 |  |  |
|  | *sul1* | Sulphonamide resistance | 661082..661921 |  |  |
|  | *mer* locus | Mercuric resistance | 664065.. 667582 |  |  |





**Fig S1. Organization of *paa* region from pSTY.** Genes are denoted by arrows. Genes, mobile elements and other features are colored based on their functional classification. Shading regions denote nucleotide identity ≥95%. Numbers in brackets indicate nucleotide positions within pSTY.


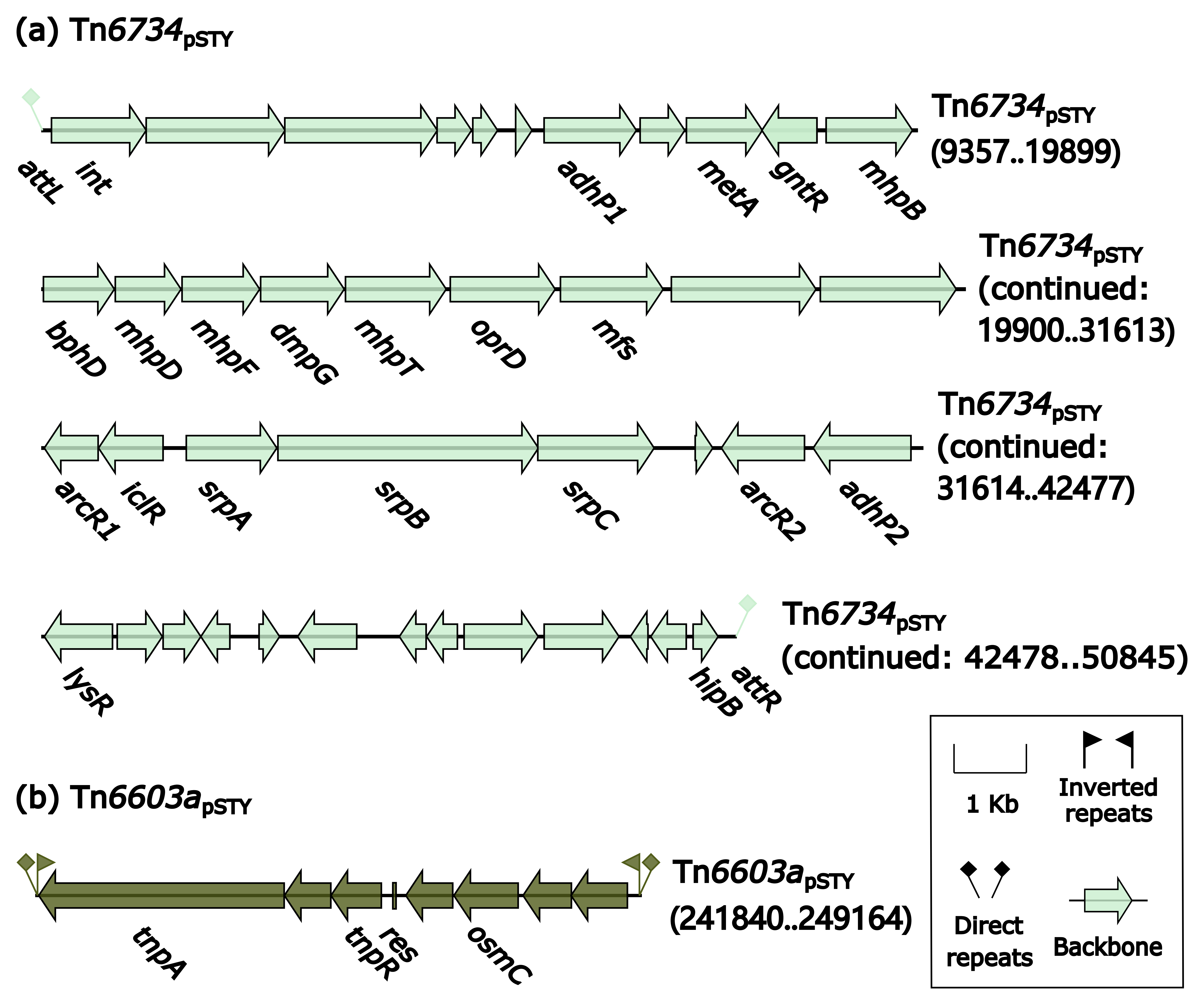


**Fig S2. Organization of Tn*6734* and Tn*6603a* from pSTY.** Genes are denoted by arrows. Genes, mobile elements and other features are colored based on their functional classification. Numbers in brackets indicate nucleotide positions within pSTY.





**Fig S3. Organization of MDR region from pJ20133-VIM, and comparison with related regions.** Genes are denoted by arrows. Genes, mobile elements and other features are colored based on their functional classification. Shading regions denote nucleotide identity ≥95%. Numbers in brackets indicate nucleotide positions within pJ20133-VIM. Accession numbers of Tn*4662a* [1] and Tn*512* [2] for reference are KJ920396 and EU306744.



**Fig S4. Organization of two Tn*7*-family elements, and comparison with related region.** Genes are denoted by arrows. Genes, mobile elements and other features are colored based on their functional classification. Numbers in brackets indicate nucleotide positions within pJ20133-VIM or p716811-VIM. Accession number of Tn*7* [3] for reference is KX117211.


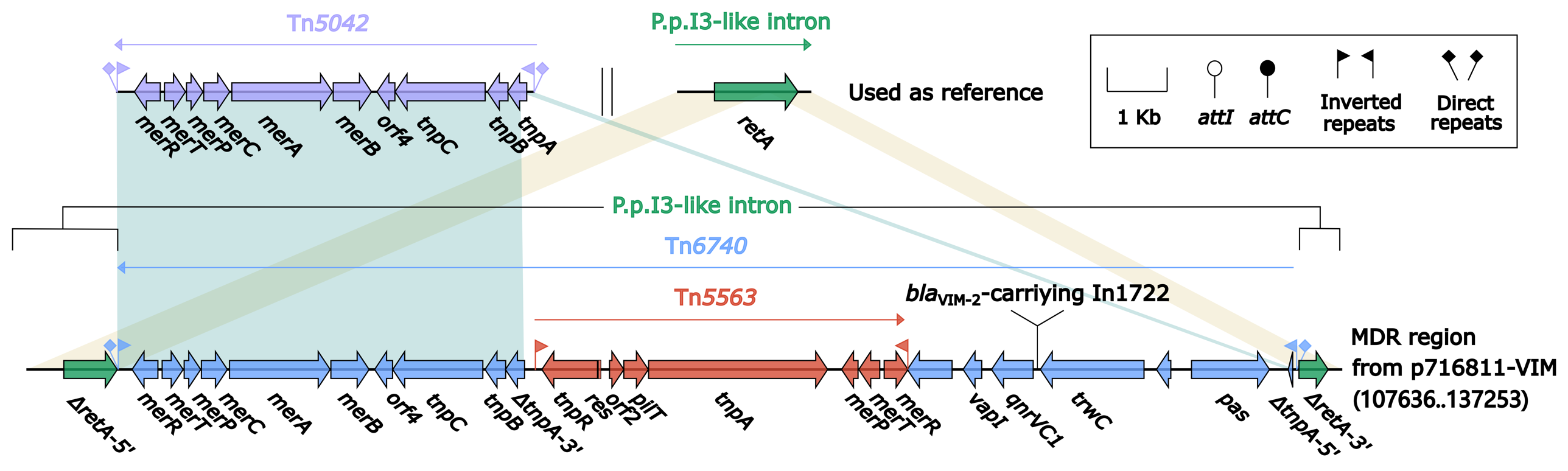
**Fig S5. Organization of MDR region from p716811-VIM, and comparison with related regions.** Genes are denoted by arrows. Genes, mobile elements and other features are colored based on their functional classification. Numbers in brackets indicate nucleotide positions within p716811-VIM. Shading regions denote nucleotide identity ≥95%. Accession number of Tn*5042* [4] for reference is AJ563380.


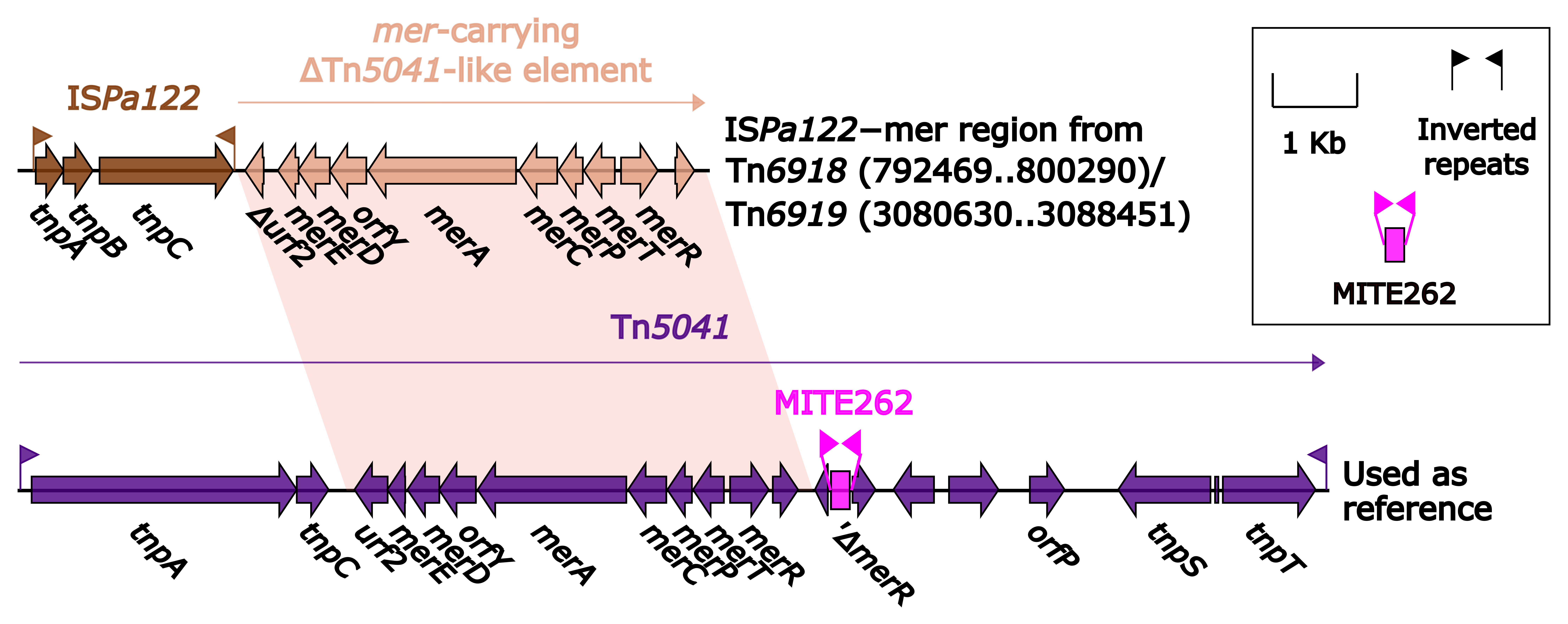
**Fig S6. Organization of IS*Pa122*−*mer* region from Tn*6918* and Tn*6919*, and comparison with related region.** Genes are denoted by arrows. Genes, mobile elements and other features are colored based on their functional classification. Shading regions denote nucleotide identity ≥88%. Numbers in brackets indicate nucleotide positions within the chromosome of strains 159349 or IEC33019. Accession number of Tn*5041* [5] for reference is X98999.



**Fig S7. Organization of *strAB* region from Tn*6919*, and comparison with related regions.** Genes are denoted by arrows. Genes, mobile elements and other features are colored based on their functional classification. Shading regions denote nucleotide identity (light blue: ≥95%; and light pink: ˂95% but ≥89%). Numbers in brackets indicate nucleotide positions within the chromosome of strain IEC33019. Accession numbers of Tn*5053* [6] and Tn*5058a* [4] for reference are L40585 and Y17897.


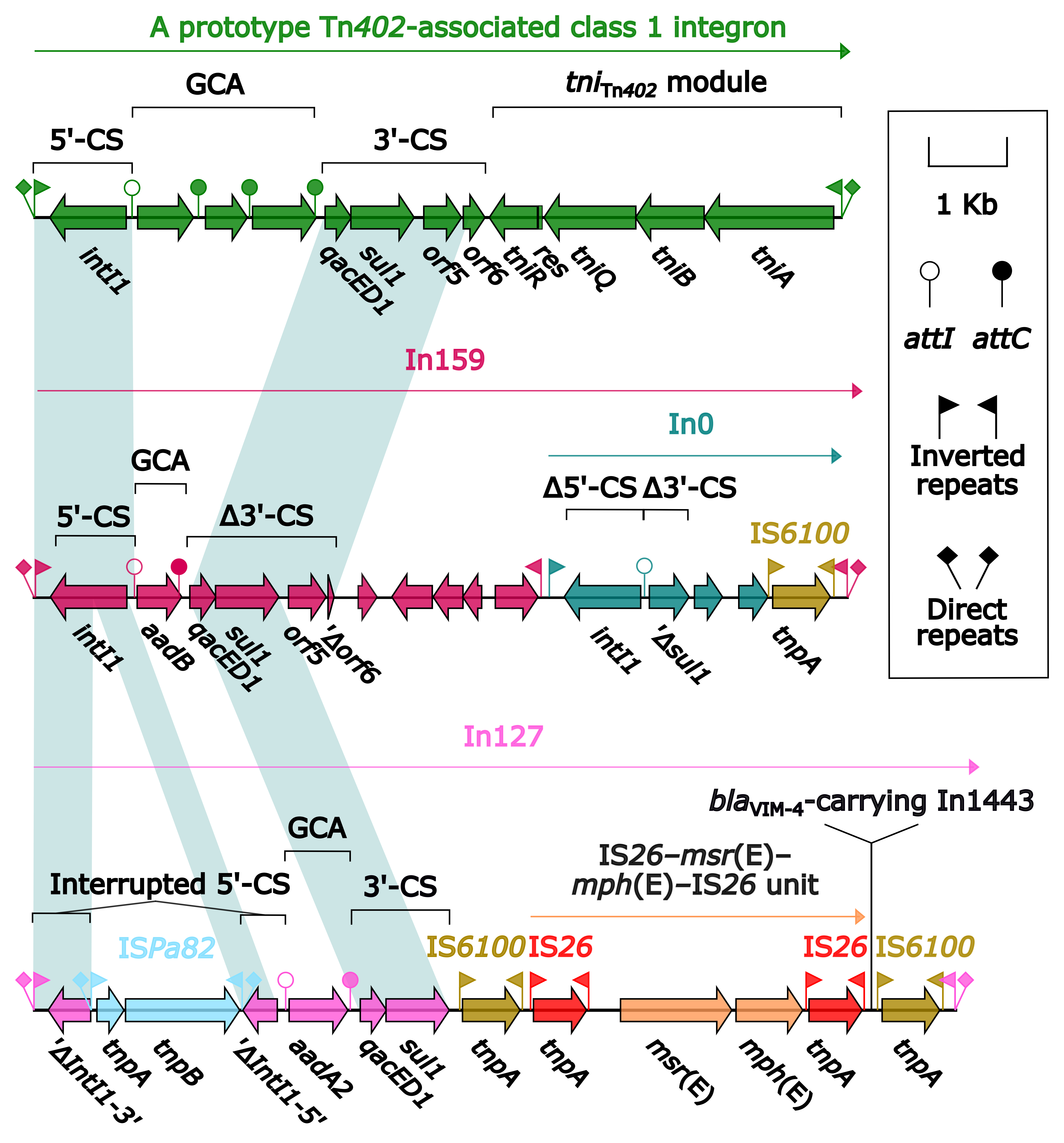


**Fig S8. Organization of In159 and In127 from Tn*6532* and Tn*6403* respectively, and comparison with related region.** Genes are denoted by arrows. Genes, mobile elements and other features are colored based on their functional classification. Numbers in brackets indicate nucleotide positions within the chromosome of strain DHS01 or 6762. Shading regions denote nucleotide identity ≥95%.

**References**

1. Szuplewska M, Ludwiczak M, Lyzwa K et al. Mobility and generation of mosaic non-autonomous transposons by Tn*3*-derived inverted-repeat miniature elements (TIMEs). PLoS One 2014; **9**: e105010.

2. Petrovski S, Stanisich VA. Tn*502* and Tn*512* are res site hunters that provide evidence of resolvase-independent transposition to random sites. J Bacteriol 2010; **192**: 1865-74.

3. Peters JE, Craig NL. Tn*7*: smarter than we thought. Nat Rev Mol Cell Biol 2001; **2**: 806-14.

4. Mindlin S, Minakhin L, Petrova M et al. Present-day mercury resistance transposons are common in bacteria preserved in permafrost grounds since the Upper Pleistocene. Res Microbiol 2005; **156**: 994-1004.

5. Kholodii G, Yurieva O, Gorlenko Z et al. Tn*5041*: a chimeric mercury resistance transposon closely related to the toluene degradative transposon Tn*4651*. Microbiology (Reading, England) 1997: 2549-56.

6. Kholodii GY, Yurieva OV, Lomovskaya OL et al. Tn*5053*, a mercury resistance transposon with integron's ends. J Mol Biol 1993; **230**: 1103-7.
